# Potent immunogenicity and broad-spectrum protection potential of microneedle array patch-based COVID-19 DNA vaccine candidates encoding dimeric RBD chimera of SARS-CoV and SARS-CoV-2 variants

**DOI:** 10.1080/22221751.2023.2202269

**Published:** 2023-05-01

**Authors:** Feng Fan, Xin Zhang, Zhiyu Zhang, Yuan Ding, Limei Wang, Xin Xu, Yaying Pan, Fang-Yuan Gong, Lin Jiang, Lingyu Kang, Zhuo Ha, Huijun Lu, Jiawang Hou, Zhihua Kou, Gan Zhao, Bin Wang, Xiao-Ming Gao

**Affiliations:** a Advaccine Biopharmaceutics (Suzhou) Co. Ltd, Suzhou, People’s Republic of China; b School of Biological Science and Basic Medicine, Soochow University, Suzhou, People’s Republic of China; c Shenzhen Qinglan Biotechnology Co. Ltd., Shenzhen, People’s Republic of China; d Changchun Veterinary Research Institute, Chinese Academy of Agricultural Sciences, Changchun, People’s Republic of China

**Keywords:** COVID-19, SARS-CoV-2, RBD chimera, DNA vaccine, microneedle

## Abstract

Breakthrough infections by SARS-CoV-2 variants pose a global challenge to COVID-19 pandemic control, and the development of more effective vaccines of broad-spectrum protection is needed. In this study, we constructed pVAX1-based plasmids encoding receptor-binding domain (RBD) chimera of SARS-CoV-1 and SARS-CoV-2 variants, including pAD1002 (encoding RBD^SARS/BA1^), pAD1003 (encoding RBD^SARS/Beta^) and pAD131 (encoding RBD^BA1/Beta^). Plasmids pAD1002 and pAD131 were far more immunogenic than pAD1003 in terms of eliciting RBD-specific IgG when intramuscularly administered without electroporation. Furthermore, dissolvable microneedle array patches (MAP) greatly enhanced the immunogenicity of these DNA constructs in mice and rabbits. MAP laden with pAD1002 (MAP-1002) significantly outperformed inactivated SARS-CoV-2 virus vaccine in inducing RBD-specific IFN-γ^+^ effector and memory T cells, and generated T lymphocytes of different homing patterns compared to that induced by electroporated DNA in mice. In consistence with the high titer neutralization results of MAP-1002 antisera against SARS-CoV-2 pseudoviruses, MAP-1002 protected human ACE2-transgenic mice from Omicron BA.1 challenge. Collectively, MAP-based DNA constructs encoding chimeric RBDs of SARS-CoV-1 and SARS-CoV-2 variants, as represented by MAP-1002, are potential COVID-19 vaccine candidates worthy further translational study.

## Introduction

Effective vaccines against infection from the severe acute respiratory syndrome coronavirus 2 (SARS-CoV-2) are crucial weapons to control the pandemic coronavirus disease 2019 (COVID-19), which has caused more than 630 million infections with more than 6.5 million deaths worldwide since late 2019 [1]. To date, more than 30 first-generation vaccines based on the ancestral (wild type, WT) strain SARS-CoV-2 and several second-generation vaccines based on SARS-CoV-2 variants of concerns (VOCs) have been approved or authorized for emergency use, including inactivated virus vaccines, viral vector vaccines, subunit vaccines and nucleic acid vaccines encoding the viral spike (S) protein [2]. Significantly decreased protective efficacies against SARS-CoV-2 variants were observed in clinical trials and real-world evidence studies of first-generation COVID-19 vaccines [2–6]. Waves of breakthrough infections of Omicron BA.5, BQ1.1 and XBB.1 around the globe in previously vaccinated individuals have been reported in recent months [7–11]. It is thus necessary to develop novel vaccines able to provide broader-spectrum protection against newly emerging SARS-CoV-2 variants.

Among the four structural proteins in SARS-CoV-2 virus, S protein is the main target for COVID-19 vaccines. It contains the receptor-binding domain (RBD) responsible for human ACE2 (hACE2) receptor binding and mediating virus entry [12,13]. Neutralizing antibodies (NAbs) specific for RBD in S1 region of the S protein play critical roles in COVID-19 protection [14,15]. Protein subunit vaccines based on recombinant WT SARS-CoV-2 RBD homodimer (RBD^WT/WT^), WT-Beta (RBD^WT/Beta^) or Delta-Omicron BA.1 (RBD^Delta/BA1^) RBD chimera induced NAb production and provided cross-protection against SARS-CoV-2 VOCs in mice and rhesus monkeys [16,17]. Tan et al. reported that BNT162b2 mRNA vaccine generated pan-Sarbecovirus NAbs in SARS-CoV survivors, suggesting that SARS-CoV-induced immunological memory cells could help production of broadly cross-reactive NAbs against SARS-CoV-2 variants [18]. It is thus reasonable to speculate that RBD chimera of SARS-CoV-1 and SARS-CoV-2 variants might function as strong immunogens able to induce broad spectrum cross-protection against SARS-related viruses.

DNA vaccines are considered an attractive alternative to conventional vaccines because they are relatively easy and inexpensive to produce, stable at room temperature, and able to stimulate balanced cellular and humoral immunity [19,20]. Several groups have explored the possibility to develop various DNA vaccines against COVID-19 with promising results. For example, COVID-eVax, an electroporated DNA vaccine candidate encoding SARS-CoV-2 RBD^WT^, elicited protective responses in animal models [21]. pGX9501, a WT SARS-CoV-2 full-length (FL) S protein-encoding electroporated DNA vaccine candidate, was able to generate NAbs as well as IFN-γ^+^ CD4^+^ and CD8^+^ T cells against WT SARS-CoV-2 as well as Delta variant in volunteers aged between 18 and 60 years [22]. In 2021, ZyCoV-D, a S protein-encoding DNA vaccine delivered by a needleless injector, was authorized for emergency use against COVID-19 in India [23]. Naked DNA plasmids, when intramuscularly (IM) or intradermally (ID) administered without assistance, are relatively poor in transfection efficiency and consequently show low level of immunogenicity in vivo [19,20]. Electroporation (EP)-, or needless injection-, assisted delivery can significantly improve DNA immunization results, but such methods often cause pain or discomfort to the vaccinees and require special expertise to operate the equipment. One possible solution to this problem is microneedle array patch (MAP) delivery which utilizes microscopic projection arrays on a plaster to deliver a vaccine in the form of a patch placed on the skin in an easy-to-use and painless fashion [24,25]. Due to its immune-rich milieu, the skin is a unique vaccination site evolutionarily primed to respond to challenges leading to strong adaptive humoral and cellular immunity [26–28]. Recent progress suggests that MAP-delivered DNA vaccines can induce satisfactory immunization results in vivo [29–31].

This study was designed to construct DNA vaccine candidates encoding heterodimeric RBDs of SARS-CoV-1 and SARS-CoV-2 variants, and then explore the possibility of developing MAP-based RBD-chimera DNA vaccines that can effectively induce cross-neutralizing Abs against antigen-matched and antigen-mismatched SARS-COV-2 VOCs. Our results support the concept that combination of the RBD chimera approach, DNA vaccination and MAP technology may open a new avenue for developing novel broadly cross-protective COVID-19 vaccines.

## Materials and methods

### Construction of DNA vaccine candidates

The synthesis of cDNA encoding heterodimeric fusion RBDs of SARS-CoV-1, prototype SARS-CoV-2 (2019-nCoV strain IVDC-HB-01/2019, GISAID: EPI_ISL_402119) and its variant B.1.351 (Beta), Omicron BA.1 and BA.5 was performed by GenScript, Nanjing, China. Optimization analysis of the cDNA sequences was performed using an in-house analytic tool, taking into accounts codon usage bias, GC content, mRNA secondary structure, cryptic splicing sites, premature poly(A) sites, internal *chi* sites and ribosomal binding sites, negative CpG islands, RNA instability motif (ARE), repeat sequences (direct repeat, reverse repeat, and dyad repeat), and restriction sites that may interfere with cloning. The resulting synthesized and optimized cDNA, together with a secretion leader peptide-encoding sequence, was cloned into expression vector pVAX1. Complete antigen-encoding DNA sequences of the 3 plasmid constructs are given in the Supplemental File SF-1. Plasmid pWT, a pVAX1-based COVID-19 vaccine candidate encoding FL S protein of SARS-CoV-2, was as previously described [32]. A pVAX1-based expression plasmid encoding FL firefly luciferase (pVAX1-Luc) was similarly constructed. Restriction enzyme analysis and DNA sequencing was performed to confirm the accuracy of construction. Plasmids were transformed into *E. Coli* strain HB101. Single colonies were undergone expansion in one-liter flasks for culturing in LB broth. Plasmids were extracted, purified by MaxPure Plasmid EF Giga Kit (Magen, China), and dissolved in distilled water at 1 mg/mL final concentration. The purity of the plasmids was measured by an agarose gel electrophoresis and a UV detector at a range of 1.8–2.0 OD260 nm/280 nm. Endotoxin contamination in plasmid samples was below 30 EU/mg by the LAL test.

### Fabrication of MAPs laden with DNA vaccine

MAPs used in this study were prepared by a two-step micro-molding process. The vaccine formulation consisting of concentrated DNA plasmid (adjusted to obtain a final dose of 20 μg per patch), water-soluble and biocompatible materials including polyvinyl alcohol (PVA), hydroxylethyl cellulose (HEC), polyvinyl pyrrolidone (PVP), sucrose and supplemental salts in 100 mM trisodium citrate buffer pH 7.4 was cast onto a PDMS mould (486 MNs per array; each cone-shaped MN measuring 450 μm in length and 160 μm in width at the base). Vacuum was applied to ensure that the formulation filled the entire MN cavity and the formulation was allowed to air dry at room temperature overnight. Then the backing formulation consisting of PVA, PVP and sucrose was cast onto the mould under vacuum and subsequently dried at room temperature for 4 h before demolding the MN patch, which was further mounted onto a 1.5 cm^2^ paper backing, followed by packaging in aluminum bags and stored at +4°C or +25°C until use. The strength of MNs was checked before and after short-term insertion in a ten-layer parafilm pack for penetration effectiveness, bending and brittleness under the microscope.

### Western blot

HEK293 cells pre-plated in a 6-well plate were transiently transfected with 2.5 μg DNA vaccine plasmids with Hieff TransTM Liposomal Transfection Reagent (YEASEN, Shanghai, China). Two days later, the cells were pelleted and lysed in lysis buffer. Cell lysates were separated by SDS-PAGE and transferred to PVDF membranes. Immunoblotting was performed by using rabbit anti-RBD^WT^ primary Ab (Bioworld, Nanjing, China) diluted 1:1,000 in 5% milk–0.05% PBS-Tween 20, and horseradish peroxidase (HRP)-labeled goat anti-rabbit IgG secondary Ab (BD Biosciences, San Diego, CA, USA). Chemiluminescence detection was performed with the ECL Prime Western Blotting System and acquired by the ChemiDoc Imaging System (Bio-Rad).

### Bioluminescence imaging

BALB/c and C57BL/6 mice (5 mice/group) were anesthetized with 97% oxygen and 3% isoflurane (Isoba, MSD Animal Health, Walton, UK) and then administered with MAP-Luc patches on shaved skin surface on dorsal sides for 15 min. Fifteen min after i.p. injection of a 15 mg/mL luciferin solution (PerkinElmer) at 10 μL/g body weight, the mice were subjected to bioluminescence imaging using IVIS Spectrum under gas anesthesia. Luciferase expression level was then quantified using the Living Image software in a fixed region of interest (ROI) in terms of photone/sec/cm2/sr.

### Animal immunization

Female BALB/c, C57BL/6 and hACE2-transgenic (C57BL/6 background) mice (6–8 weeks of age) were purchased from Shanghai Slac Laboratory Animal Co., Ltd. and maintained under SPF conditions at the animal facilities of Advaccine Biologics (Suzhou) Co. New Zeeland white rabbits, purchased from Shanghai Somglian Experimental Animal Company, were housed in the Grade I animal facilities of Advaccine Biologics (Suzhou) Co. All animal experiments were performed in compliance with the recommendations in the Guide for the Care and Use of Laboratory Animals of the Ministry of Science and Technology Ethics Committee and approved (document No. 2021070102) by on the Ethics Committees of the company.

EP-assisted DNA immunization was performed by IM inoculation of DNA (1 mg/mL in SSC, 20 μg for mice, 200 μg for rabbits) followed by EP using Inovio CELLECTRA®2000 and a 3P array (Inovio, San Diego, CA, USA) with two sets of pulses with 0.2 Amp constant current. All IM + EP-delivered vaccines were primed on day 0 and boosted on day 14 unless otherwise indicated. Blood samples were collected on days 0, 14, 21 and/or 28.

To administer MAP-based DNA vaccines in mice (1 patch/mouse) and rabbits (10 patches/rabbit), larger than patch-sized skins on dorsal sides were shaved and treated with hair removal cream one day earlier. Apply and slightly press the patch with thumb pressure on the shaved skin surface of the animals under anesthesia, allow to stay for 15 min and then peel off the used MAP. MAPs fabricated in this study did not cause skin allergy or physical damage, and within 6 days fur of the shaved skins returned to normal.

### Authentic SARS-CoV-2 virus and in vivo efficacy in a mouse model

The authentic SARS-CoV-2 Omicron BA.1 (SARS-CoV-2 strain Omicron CoV/human/CHN_CVRI-01/2022) strain was isolated from COVID-19 patients. Groups of hACE2-transgenic mice that had been immunized with MAP-1002, pAD1002/IM + EP, or pVAX1/IM + EP, were anesthetized and intranasally inoculated with 5 × 10^3^ TCID50 of SARS-CoV-2 Omicron BA.1 variant. Their body weight was measured daily, and the mice were euthanized at 4 days post-infection (dpi) to harvest lung tissues for virological assessment or histological examination. All experiments associated with the authentic viruses were conducted in Biosafety Level 3 laboratory with standard operating procedures.

### RT-qPCR

SARS-CoV-2 RBD-specific quantitative reverse transcription-PCR (RT-qPCR) assays were performed by using a FastKing One Step Probe RT-qPCR kit (Tiangen Biotech, China) on a CFX96 Touch real-time PCR detection system (Bio-Rad, USA). In efficacy studies, SARS-CoV-2 viral RNA in lung tissue homogenates was extracted using a QIAamp Viral RNA Kit (QIAGEN, Hilden, Germany). Virus copy numbers were determined by RT-qPCR, using a HiScript II One Step qRT-PCR SYBR Green Kit (Vazyme Biotech, Nanjing, China). Primers were designed based on the N and sgE gene sequences of SARS-CoV-2, and the viral RNA load in the lung tissues was determined by the TaqMan fluorescent quantitative PCR method as we previously described [33].

### Enzyme-linked immunosorbent assay

Antibody titration was performed on sera obtained by retro-orbital bleeding from mice or venous bleeding from the ears of rabbits. The ELISA plates were functionalized by coating with the recombinant RBD proteins (SinoBiological, Beijing, China) at 1 μg/mL and incubated 18 h at 4°C and subsequently blocked with 3% BSA-0.05% Tween 20-PBS (PBST) for 1 h at room temperature. Serially diluted serum samples were then added in triplicate wells, and the plates were incubated for 1 h at room temperature. After a double wash with PBST, horse-radish peroxidase (HRP)-conjugated Ab against murine (Abcam, ab6789, 1/2000 diluted), or rabbit (GenScript, A00098, 1:2,000 diluted) IgG was added and then developed with 3,3′,5,5′-tetramethylbenzidine (TMB) substrate (Coolaber, CN). The reaction was stopped with 2 M of H_2_SO_4_, and the absorbance measured at 450 nm and reference 620 nm using a microplate reader (TECAN, CH).

### Neutralization antibody detection

The pseudovirus microneutralization assay was performed to measure neutralizing antibody levels against prototype SARS-CoV-2 and its variants. VSV-based pseudovirus stocks of prototype SARS-CoV-2 and B.1.35, P.1, B.1.617.2 variants were purchased from Gobond Testing Technology (Beijing, China), which were aliquoted for storage at −80°C. hACE2 stable expressing HEK293T cells (prepared in our lab) were used as target cells plated at 10,000 cells/well. SARS-CoV-2 pseudo-viruses were incubated with heat-inactivated (56°C for 30 min) and 1/3 serial diluted mouse sera for 90 min at room temperature; then, the sera-pseudovirus mixtures were added to hACE2-HEK293T cells and allowed to incubate in a standard incubator 37% humidity, 5% CO2 for 72 h. The cells were then lysed using Bright-Glo™ Luciferase Assay (Promega Corporation, Madison, WI, USA), and RLU was measured using an automated luminometer. Fifty percent pseudovirus neutralization titer (pVNT50) was determined by ﬁtting nonlinear regression curves using GraphPad Prism and calculating the reciprocal of the serum dilution required for 50% neutralization of infection. These assays have been performed in a BSL-2 facility of Advaccine. Pseudovirus neutralization experiments using Vero cells were contracted to Gobond Testing Technology, Beijing, China.

### ELISpots

Spleens and draining lymph nodes (LNs) from immunized mice were collected and used to prepare single cell suspension in RPMI-1640 medium supplemented with 10% FBS and penicillin/streptomycin. ELISpot was performed using mouse IFN-γ and IL-4 ELISpot PLUS kits (MABTECH, Cincinnati, OH, USA) according to the manufacturer’s protocol. Briefly, 5 × 10^5^ freshly prepared mouse splenocytes, or LN cells, were plated into each well and stimulated for 20 h with pooled overlapping 15-mer peptides (10 μg/ml) covering respective RBDs at 37°C in a 5% CO_2_ incubator. PMA/Iono was used for positive controls. The plates were processed in turn with a biotinylated detection antibody. Spots were scanned and quantified using AID ImmunoSpot reader (AID, Germany). IFN-γ- and IL-4-spot forming units were calculated and expressed as SFUs per million cells.

### Flow cytometry

For Intracellular cytokine staining (ICS), freshly isolated mouse splenocytes or LN cells were stimulated with an overlapping peptide pool of RBD^WT^ (10 μg/mL) in the presence of Brefeldin A (Invitrogen, USA) for 5 h at 37°C, 5% CO_2_. The cells were harvested and stained with anti-CD3, anti-CD4 and anti-CD8α surface markers, and subsequently fixed and permeabilized in permeabilizing buffer (eBiosciences, USA) and stained with fluorescence-conjugated anti-IFN-γ, anti-TNF-α, anti-IL-2 and anti-IL-4 antibodies.

For virus-specific CTL frequency analysis, PE-labeled H-2D^b^ tetramer containing the “S” epitope (SVLYNSASF) of RBD (HELIXGEN, Guang Zhou, China) was used. Freshly prepared mouse splenocytes or LN cells were stained with anti-CD45-AF700, CD3-FITC, CD8-APC,PE-tetramer, followed by FACS analysis. All fluorescence-labeled Abs were from BioLegend, and the stained lymphocytes were analysed on Attune NxT Flow Cytometer (ThermoFisher, USA).

### Molecular structure AI modelling

AlphaFold2 was used for structure predictions with the required homology modelling databases running on ColabFold. The pLDDT plots generated and the obtained structures were further visualized by PyMol 2.4.

### Statistics

Statistical analyses were performed with GraphPad Prism software version 9 (GraphPad). Error bars indicate the standard error of the mean (SEM). We used Mann–Whitney t-tests to compare two groups with non-normally distributed continuous variables and two-way ANOVA followed by Sidak’s multiple comparisons tests to analyse experiments with multiple groups and two independent variables. Significance is indicated as follows: *p < 0.05; **p < 0.01. Comparisons are not statistically significant unless indicated.

## Results

### Preparation and immunogenicity evaluation of DNA constructs encoding RBD fusion chimera of SARS-CoV-1 and SARS-CoV-2 variants

**Figure 1.**
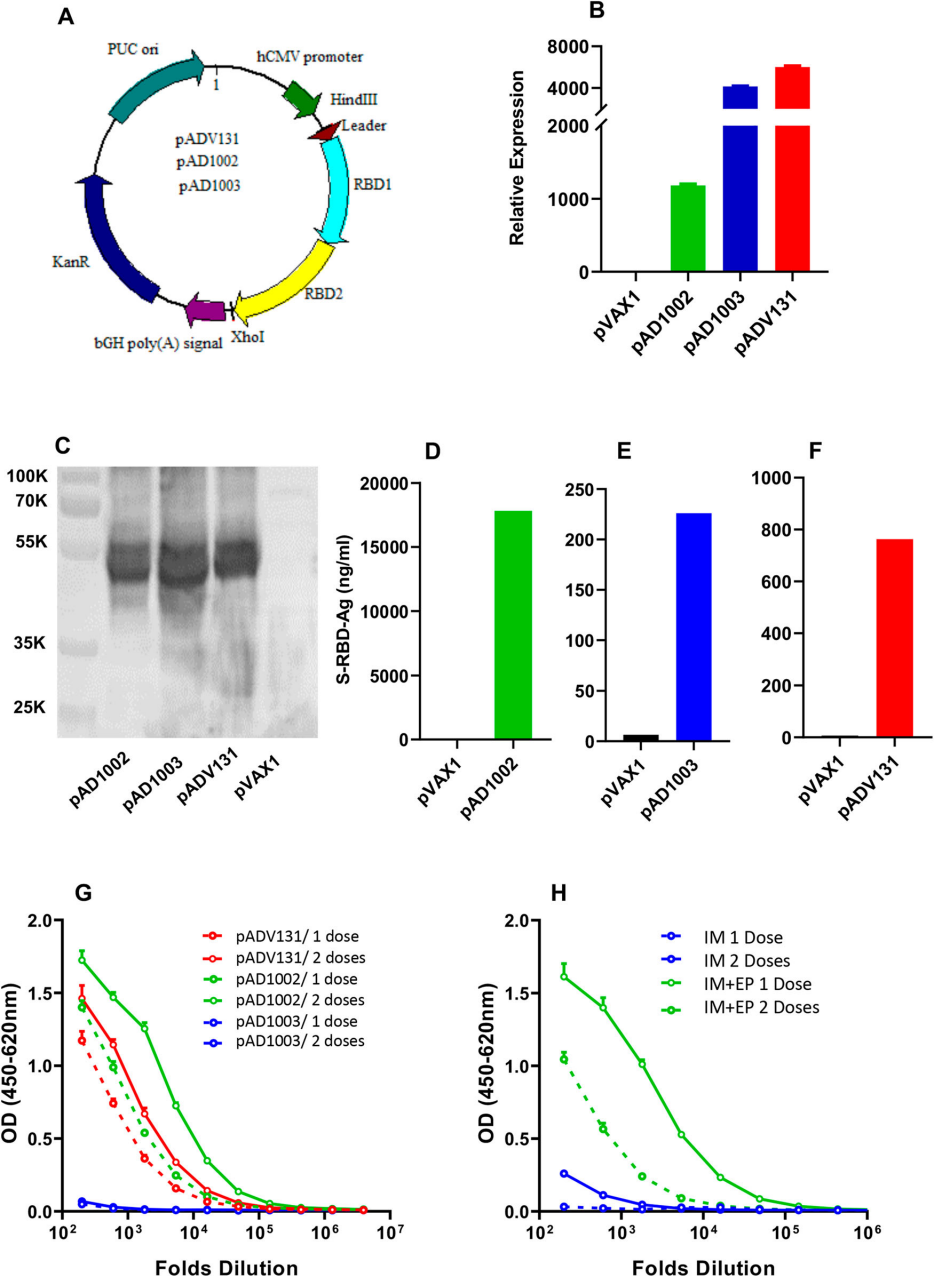
Construction and immunogenicity evaluation of COVID-19 vaccine candidates. (**A**) Schematic diagram showing the structure of pVAX1-based vaccine candidates encoding heterodimeric fusion RBDs of SARS, Beta and Omicron BA1 with a secretion leader sequence. Gene fragments were cloned into the BamH1 and XhoI sites of pVAX1 vector under human CMV promoter control. HEK293T cells, transiently transfected with pVAX1, pAD1002, pAD1003, or pADV131 24h earlier, were lysed, and the lysate subjected to (**B**) qPCR detection of RBD mRNA transcripts using GPDH as internal control, and (**C**) SDS-PAGE gel electrophoresis followed by Western blotting using anti-RBD^WT^ Abs for detection. Culture supernatant of the transfectant HEK293T cells was harvested for quantitation of secreted recombinant RBD chimera using RBD^WT^-based ELISAs (**D–F**). (**G**) Serum samples, collected from BALB/c mice after primary (1 dose) and secondary (2 doses) IM immunizations with plasmid pAD1002, pAD1003 or pADV131 (20 μg/dose) were titrated against recombinant RBD^WT^ in ELISAs. (**H**) Sera from BALB/c mice 14 days after primary (1 dose) and secondary (2 doses) IM, or IM + EP, inoculation of plasmid pWT were analysed in RBD^WT^-based ELISA. Data represent mean ± SD (*n* = 3 biologically independent samples).

Three pVAX1-based COVID-19 vaccine candidates encoding heterodimeric fusion RBDs between SARS-CoV-1 (GenBank accession no: AY278488.2), SARS-CoV-2 variant Beta (EPI_ISL_860630, GISAID) and Omicron BA.1 (EPI_ISL_6640917, GISAID), namely pAD1002 (encoding RBD^SARS/BA1^), pAD1003 (encoding RBD^Beta/BA1^) and pADV131 (encoding RBD^SARS/Beta^), respectively, were constructed (Figure 1(A)). RNA- and codon-optimization was performed to increase the expression efficiency of the DNA constructs in mammalian cells. To promote protein secretion, we introduced a unique secretion leader sequence in the fusion RBD constructs. FL amino acid sequences of the antigens encoded by the 3 vaccine constructs are presented in supplemental file **SF-2**, highlighting the secretion leader and fusion RBD sequences. No linker sequence was added between the fusion RBDs. Expression of these plasmids in transfected HEK293T cells was confirmed by qPCR and Western blotting (Figure 1(B,C)). Secreted recombinant RBD proteins were readily detectable in culture supernatant of the transfectant cells by ELISAs (Figure 1(D,F)). The level of qPCR-detected RBD mRNA transcription in pAD1002-tranfected HEK293 cells was several folds lower than that of the pAD1003-, or pAD131-, transfectants (Figure 1(B)). This, however, did not result in reduced intracellular expression, or secretion, of the recombinant pAD1002 RBD protein (Figure 1(C–F)). For immunogenicity evaluation, groups of BALB/c mice were IM administered with 2 doses (20 μg/dose, with fortnight intervals) of the plasmids, followed by ELISA monitoring of serum IgG against recombinant SARS-CoV-2 RBD^WT^. As shown in Figure 1(G), pAD1002 and pADV131 induced reasonably strong IgG responses in mice, whilst pAD1003 was essentially non-immunogenic. In an earlier study, we characterized a SARS-CoV-2 FL S protein-encoding construct, pWT, which required EP assistance to trigger decent humoral responses in vivo [32]. Like pAD1003, plasmid pWT was unable to elicit RBD-specific IgG production in mice when IM administered without EP (Figure 1(H)). Given that pAD1002 and pADV131 differ from pAD1003 and pWT in possessing SARS-CoV-1 RBD (RBD^SARS^)-encoding sequence, these results argue for a potent immunogenicity-boosting effect of RBD^SARS^ in vivo.

### Fabrication and characterization of MAPs for intradermal DNA delivery

**Figure 2.**
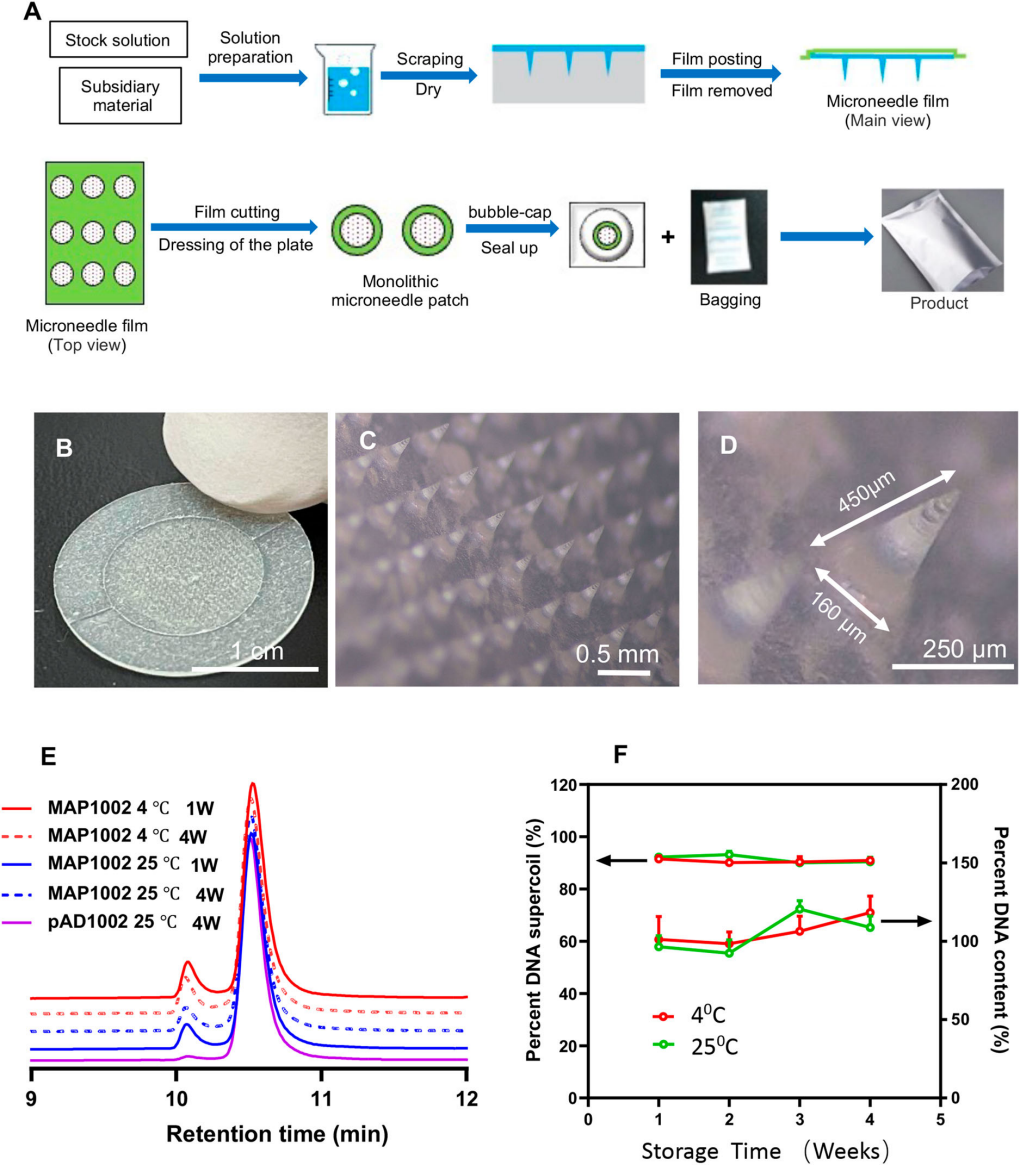
Fabrication of MAP^Adv^ for intradermal DNA delivery. (**A**) Schematic diagram showing the fabrication procedure for MAP^Adv^. (**B**) Photograph of a MAP^Adv^ skin patch, which is of 1.2 cm in diameter, with an array of 486 MNs loaded with 20 μg plasmid DNA. (**C, D**) Microphotographs show the structure of arrayed MNs in MAP^Adv^. The MNs in MAP^Adv^ patch are 450 μm in height, including a 350 μm cone-shaped needle and tapered base of 100 μm (**D**). For stability analysis, samples of MAP-1002 patches, maintained at 4°C or 25°C for 1–4 weeks, were emersed in 2 ml saline for 1 h to allow complete dissolution of MNs. The solution was subjected to HPLC profiling (**E**) and quantitation of DNA (**F**), expressed as percent plasmid DNA in supercoil form (left axis) and percent DNA recovery (right axis).

We next sought to develop novel COVID-19 vaccine preparations by combining the RBD chimera-encoding DNA constructs with dissolvable MAP technology, which may bypass the need of EP or needleless injection for satisfactory DNA vaccine immunization. The two-step micro-molding procedure to fabricate Advaccine MAP (MAP^Adv^) is shown in Figure 2(A), which has consistently given sharp and robust MN structures capable of penetrating stratum corneum with thumb pressure. The resulting round-shaped skin patch is arranged in a 486 MN array covering an area of 1.5 cm^2^ (Figure 2(B)). Microscopic examination confirmed that the arrayed MNs are 550 μm in height, including 450 μm cone-shaped needle and 100 μm base. DNA plasmids are entrapped in the top third region of the dissolvable MNs (20 μg plasmid DNA per patch), the width of pinpoint is less than 10 μm and the tapered base 160 μm (Figure 2(C,D)). Once inside the epidermis, MN tips readily dissolve to release the DNA load within minutes. The individually bagged MAP^Adv^ patches laden with pAD1002, pAD1003 or pADV131 (namely MAP-1002, MAP-1003 and MAP-131, respectively) were structurally and functionally stable at room temperature. After 30 days of storage at 25°C, for example, over 98.5% plasmid DNA recovered from MAP-1002 remained in a supercoiled form (Figure 2(E,F)), and the MAP retained potency for intradermal immunization (see below).

### Intradermal expression of luciferase gene delivered using MAP^Adv^

**Figure 3.**
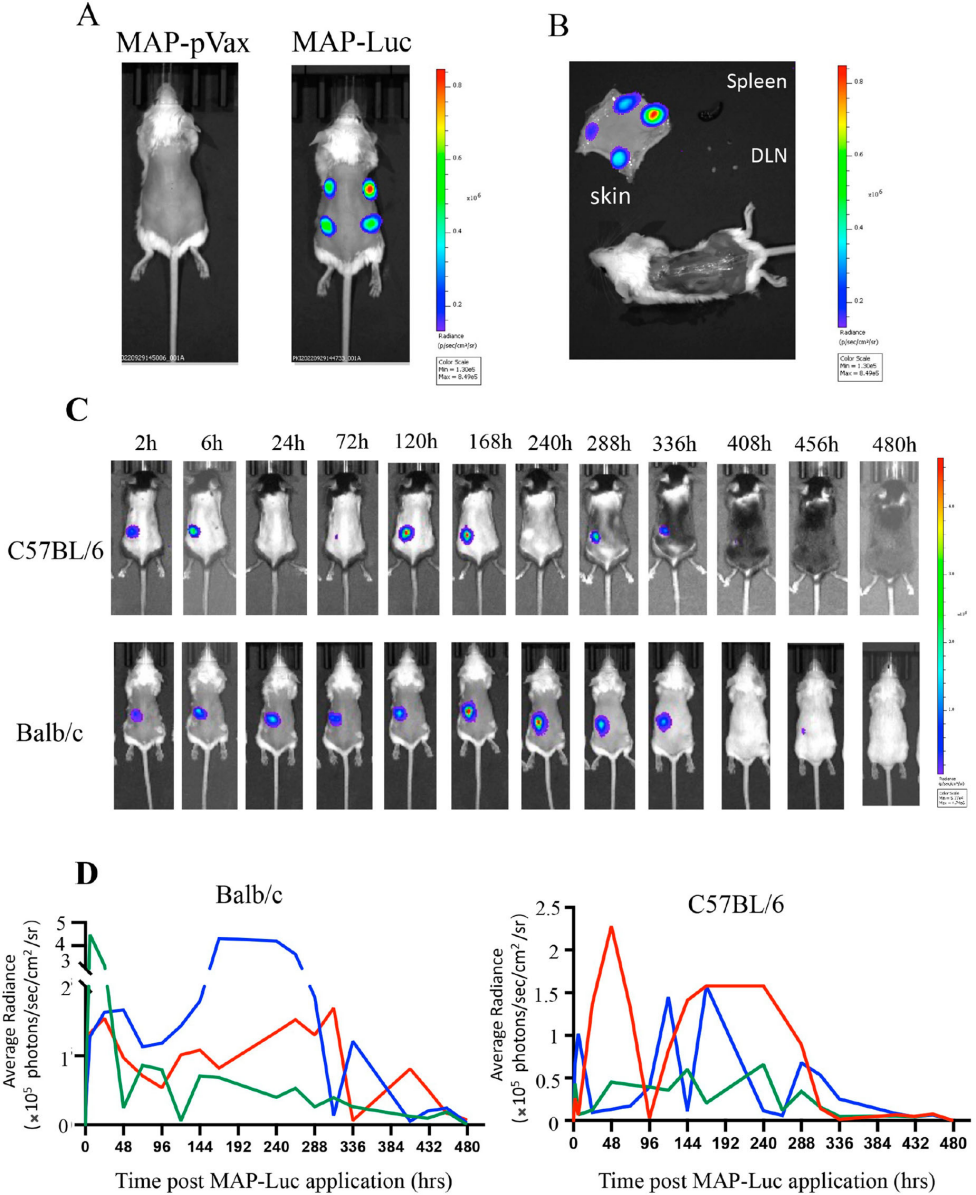
In vivo visualization of gene expression in mouse skin after MAP^Adv^-mediated pVAX1-Luc delivery. (**A**) Bioluminescent images (automated exposure) of living BALB/c mice administered with 4 MAP-Luc, or MAP-pVAX1, patches on dorsal sides 24 h previously. (**B**) Freshly prepared dorsal skin, skinned body, dLNs and spleen from a BALB/c mouse treated with 4 MAP-Luc patches 48 h earlier were subjected to bioluminescent imaging. (**C, D**) C57BL/6 and BALB/c mice (*n* = 3) were administered with MAP-Luc patches (1 patch each mouse, on dorsal side, for 15 min), followed by bioluminescent imaging at different timepoints up to 480 hrs post administration. Images from one representative mouse of each group are shown in **C**. (**D**) Bioluminescence intensity at sites of MAP-Luc application for each C57BL/6 or BALB/c mouse throughout the 480 hrs observation period was quantitated and recorded. Each line represents one individual mouse. The above is representative of 3 independent experiments.

Luciferase (Luc) activity in living animals can be visualized by bioluminescence imaging in the presence of D-luciferin. To assess the expression efficiency of MAP^ADV^-delivered genes in vivo, we prepared a pVAX1-based construct encoding FL firefly Luc (pVAX1-Luc), and then fabricated MAPs laden with 20 μg pVAX1-Luc plasmid DNA (MAP-Luc). For initial functional evaluation, BALB/c mice were treated with 4 MAP-Luc patches on separate spots of dorsal sides, followed by bioluminescence imaging 24 h thereafter. Strong bioluminescence signals at all MAP-Luc application sites confirmed high level expression of the MAP-delivered Luc gene (Figure 3(A)). The mice were sacrificed 48 h after MAP-Luc treatment for skins, skinned bodies, spleens and draining LNs (dLNs) which were immediately subjected to bioluminescence imaging. Bioluminescence signals were detected on patch application sites on the skins, but not the skinned bodies, spleens or dLNs (Figure 3(B)), confirming the retention and expression of MAP-delivered Luc gene within dermis layers.

To study the expression kinetics of MAP-delivered DNA in vivo, groups of BALB/c and C57BL/6 mice were treated with MAP-Luc patches (*n* = 3, 1 patch/mouse) for 15 min and then monitored for Luc gene expression (biofluorescence) at different timepoints until Day 20. As illustrated in Figure 3(C), strong bioluminescence signals were readily detectable at the MAP-Luc patch application sites as early as 2 h post administration in both strains of mice, which lasted in waves for up to 15 days (Figure 3(D)). The strong and durable intradermal expression of MAP-delivered Luc gene in mice foretold sound immunogenicity for the MAP-based DNA vaccine candidates.

### Immunogenicity-boosting effect of MAP^Adv^ in DNA immunization

**Figure 4.**
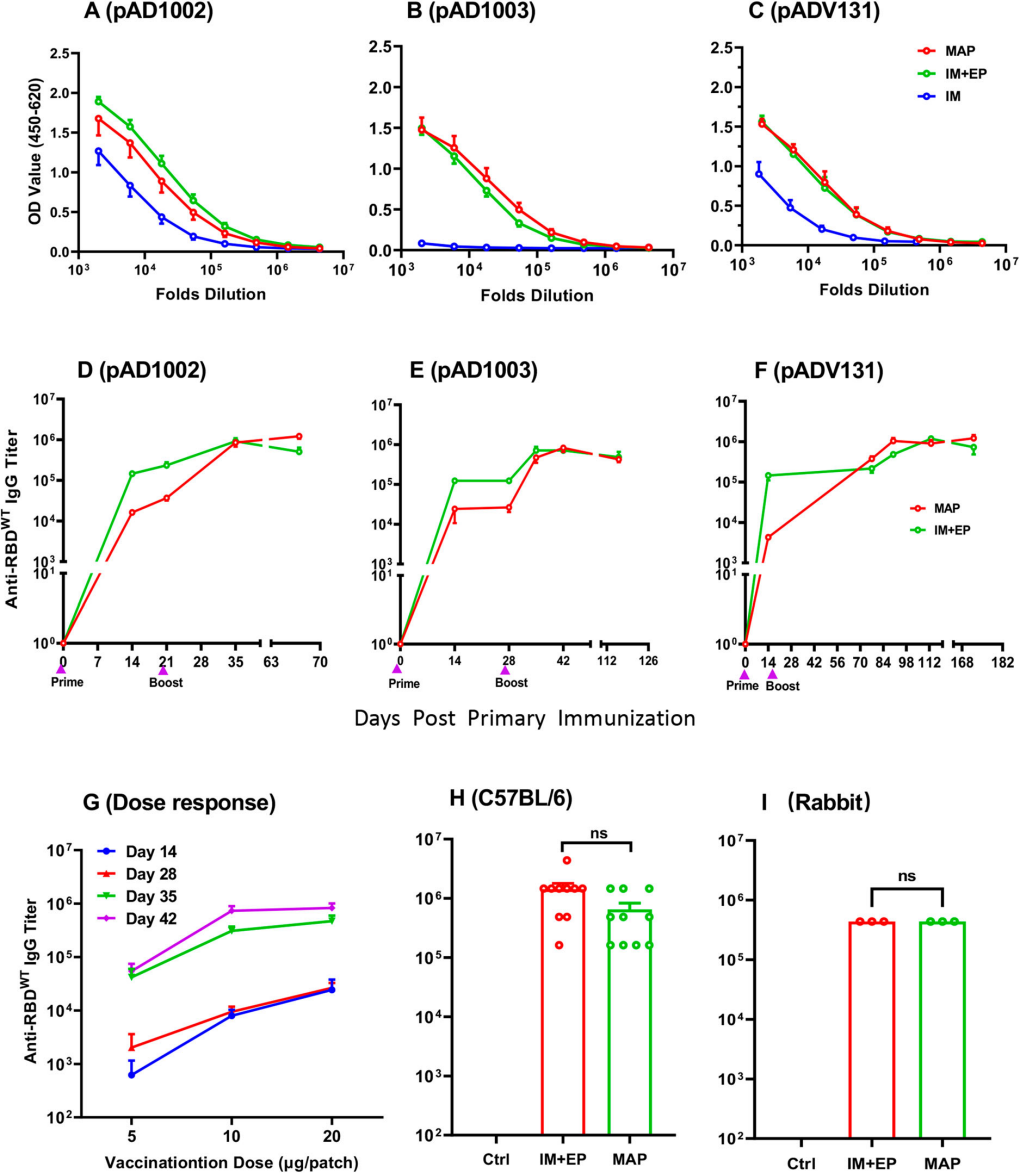
Immunogenicity-boosting effect of MAP^Adv^ in DNA immunization. Groups of BALB/c mice were given two doses (20 μg DNA/dose, with fortnight intervals) of plasmid pADV1002 (**A**), pAD1003 (**B**), or pADV131 (**C**) via MAP, IM or IM + EP delivery. Serum samples, collected 14 days after boost, were titrated against recombinant RBD^WT^ in ELISAs. Serum IgG titers in BALB/c mice after MAP- or IM + EP-mediated immunization with plasmid pAD1002 (**D**), pAD1003 (**E**), or pADV131 (**F**) were monitored using RBD^WT^-based ELISAs for up to 70, 126, or 182 days, respectively, after primary immunization. (**G**) Serum samples, collected from BALB/c mice (*n* = 5) on days 14, 28, 35 and 42 after primary (Day 0) immunization with decreasing DNA doses (20, 10, 5 μg/dose) delivered by a full-, half- or quarter-sized MAP-131, respectively, were titrated against recombinant RBD^WT^ in ELISAs. Boost immunization was given with the same dosage MAP-131 on Day 14. Dose-response curves were obtained by plotting endpoint dilution serum IgG titers against MAP-delivered DNA doses. (**H**) Serum IgG titers in C57BL/6 mice (*n* = 5) and (**I**) New Zeeland white rabbits (*n* = 3) administered with two doses of MAP-1002 (1 patch/dose/mouse, 10 patches/dose/rabbit), or pAD1002/IM + EP (20 μg/dose/mouse, 200 μg/dose/rabbit), or pVAX1/IM + EP as control. Data represent mean ± SEM.

EP has so far been regarded as the most effective, albeit rather uncomfortable, method to facilitate DNA vaccine immunogenicity. To assess if MAP^Adv^ could be as efficient as EP in enhancing DNA vaccination results, BALB/c mice were administered with two doses of MAP-1002, MAP-1003 or MAP-131 (1 patch/dose, with a fortnight interval). For controls, two doses of corresponding plasmids (20 μg/dose) were IM injected with, or without, EP. By day 14 post boost, serum samples from the MAP, IM and IM + EP groups were titrated against RBD^WT^ in ELISAs for comparison. As shown in Figure 4(A–C), MAP and IM + EP delivery of plasmid pAD1002 and pADV131 led to approximately 5 folds higher anti-RBD IgG titers compared to IM immunization, and there was no significant difference between the MAP and IM + EP groups. The significant MAP enhancement of DNA immunization is best demonstrated by comparing the data of the pAD1003/IM and MAP-1003 groups. MAP-1003 completely overcame the very poor immunogenicity of IM-inoculated plasmid pAD1003 in vivo (Figure 4(B)). RBD^WT^-binding serological IgG titers in the MAP^Adv^ and IM + EP groups were monitored until 70 (Figure 4(D), pAD1002), 126 (Figure 4(E), pAD1003) or 182 (Figure 4(F), pAD131) days after boost, no significant Ab titer decline was found in these groups throughout the periods of observation. Dose–response curves of MAP-mediated DNA vaccination were obtained by plotting RBD^WT^-binding IgG titers against decreasing DNA doses delivered by a full-, half-, or quarter-sized MAP-131 (Figure 4(G)). Additionally, MAP-1002 was also employed to immunize C57BL/6 mice (1 patch/dose/mouse) and New Zeeland white rabbits (10 patches/dose/rabbit), with MAP-pVAX1 and pAD1002/IM + EP as controls. Similarly strong and lasting IgG responses to MAP-1002 and pAD1002/IM + EP immunizations were observed in both species of animals (Figure 4(H,I)). Thus, MAP^Adv^ may represent a potential alternative to IM + EP for facilitating DNA vaccination in vivo.

### Virus-specific CTL responses elicited by MAP-1002 in mice

**Figure 5.**
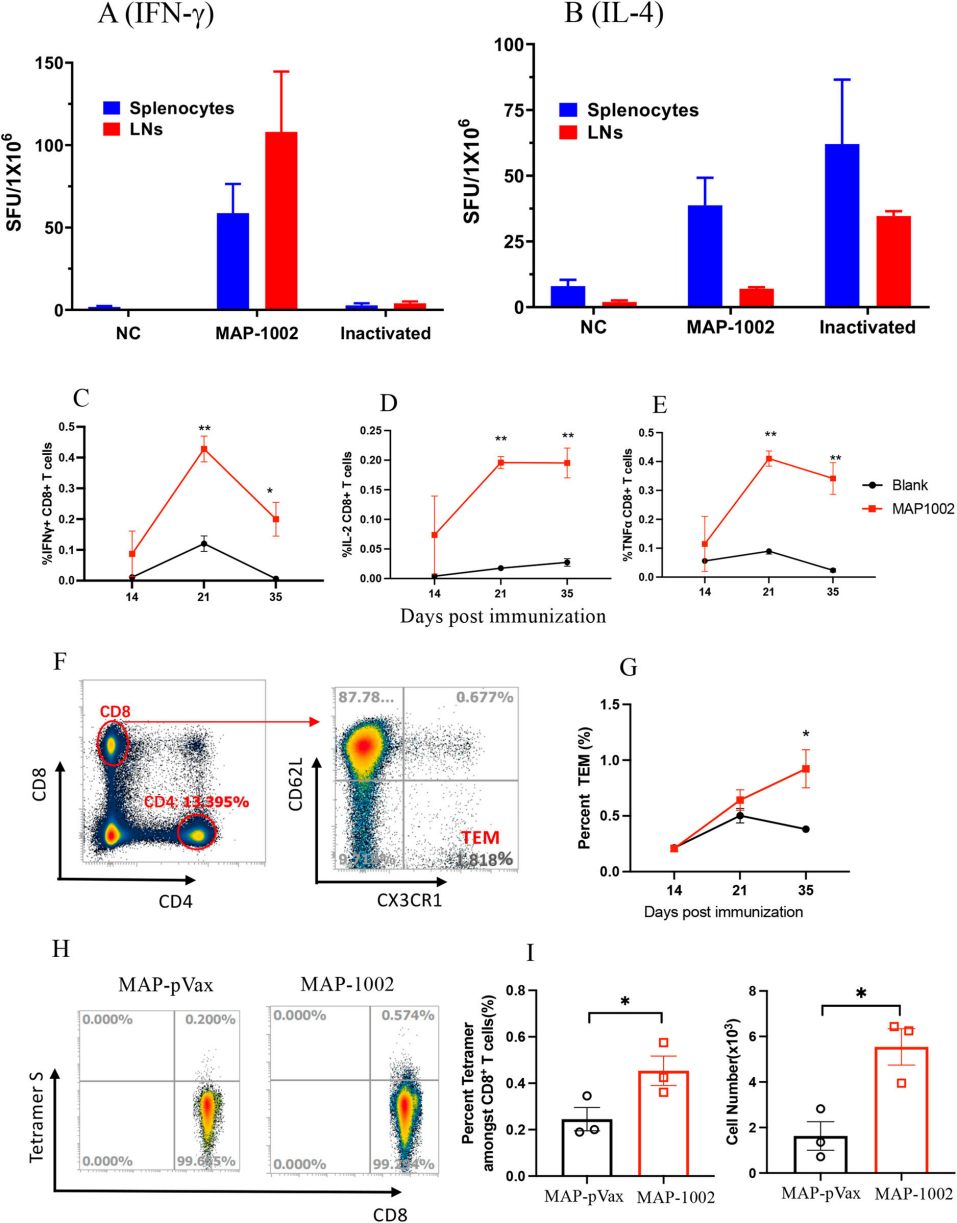
SARS-CoV-2-specific T cell responses induced by MAP-1002 in mice. BALB/c mice were immunized twice with, or without (NC), MAP-1002 or inactivated virus vaccine (Inactivated) and then sacrificed for spleens and dLNs 14 days after boost. ELISpot analyses of (**A**) IFN-γ and (**B**) IL-4 spot-forming cells (SFC) in splenocytes and dLN cells were performed after re-stimulation with pooled 14-mer overlapping peptides spanning the RBD^WT^ sequence. (**C–E**) CD8^+^ T cells amongst dLN cells of BALB/c mice 14, 21 and 35 days post MAP-1002 immunization were assayed for IFN-γ (**C**), IL-2 (**D**) and TNF-α (**E**) expression by intracellular cytokine staining (ICS) and FACS analysis after re-stimulation with the RBD^WT^ peptide pool. LN cells from the unimmunized (Blank) mice were included as control. (**F**) Gating strategy for CD8 ^+ ^CD62L^-^CXCR1^+^ TEMs in mouse LN cells. (**G**) Percentages of CD8^+^ TEM amongst LN cells of BALB/c mice 14, 21 and 35 days after MAP-1002 administration (red line), as revealed by flow cytometric analysis results, are compared with that of unimmunized mice (black line). (**H**) Draining LN cells from C57BL/6 mice 14 days after MAP-1002, or MAP-pVAX1, immunization were stained with PE-labeled “S Tetramer” for identification of CD8^+^ T cells expressing TCR specific for S peptide/H-2D^b^ complex by flow cytometry. (**I**) Histograms compare the percentages (left panel), or absolute numbers (right panel), of the *S* tetramer^+^ cells amongst CD8^+^ T lymphocytes of C57BL/6 mice immunized with either MAP-pVAX1 or MAP-1002. Data are means ± SEM. *P* values were analysed with two-tailed Mann-Whitney test (*p < 0.05; **p < 0.01).

Compared to inactivated virus or subunit viral protein vaccines, nucleic acid vaccines are particularly powerful in generating MHC I-restricted CD8^+^ cytotoxic T lymphocytes (CTL) known to play pivotal roles in protection against viral infections in vivo [20]. SARS-CoV-2-specific CTL responses have been found to be associated with milder situations in acute and convalescent COVID-19 patients [34]. To evaluate the ability of MAP-DNA vaccine candidates to induce CTLs in vivo, C57BL/6 mice were administered with 2 doses of MAP-1002, or inactivated WT SARS-CoV-2 vaccine, or IM + EP-delivered pVAX1 as control, followed by ELISpot detection of RBD-responding IFN-γ^+^ and IL-4^+^ cells in peripheral lymphoid organs. Interestingly, RBD-responsive IFN-γ^+^ cells were found in dLNs and, to a lesser extent, spleens of mice immunized with MAP-1002, but not pVAX1 or inactivated virus vaccine (Figure 5(A)). On the other hand, IL-4^+^ cells elicited by MAP-1002 were found mostly in spleens rather than dLNs, while IL-4^+^ cells generated by inactivated virus vaccine were detectable in both spleens and LNs (Figure 5(B)).

Intracellular cytokine staining (ICS) results confirmed that RBD-specific IL-2-, IFN-γ− and TNF-α-expressing CD8^+^ T cells were clearly identifiable in dLNs from BALB/c mice 14, 21 and 35 days post MAP-1002 immunization (Figure 5(C–E)). By Day 35, the percentage of CD8^+^ T cells bearing CX3CR1, a surface marker for effector memory CD8^+^ T cells (TEM), in dLNs of MAP-1002-immunized mice were significantly higher compared to that of the pAD1002/IM or unimmunized control groups (Figure 5(F,G)). EP-assisted DNA immunization is well known for ability to trigger strong T cell immunity in vivo [20]. In contrast to the “dLN-favoring” distribution pattern of MAP-1002-induced RBD-specific T cells, however, pAD1002/IM + EP immunization of BALB/c mice generated RBD-responsive IFN-γ^+^ cells almost exclusively present in the spleens rather than dLNs (**supplemental Fig. S1**).

Several mouse CTL epitopes have been identified in the amino acid sequence of SARS-CoV-2 RBD, including a H-2D^b^-restricted “S” epitope (amino acid residues 366–374, SVLYNSASF) [35]. We employed the *S* tetramer (PE-labeled H-2D^b^ tetramer harbouring the “S” peptide), in flow cytometric analysis, to trace CD8^+^ T lymphocytes bearing TCRs capable of recognizing the *S* epitope amongst dLN cells from MAP-1002-primed C57BL/6 mice. As illustrated in Figure 5(H,I), the LN-residing S epitope-specific CTLs expanded significantly as result of MAP-1002 immunization in C57BL/6 mice. Collectively, the above data indicate that MAP-mediated DNA immunization can generate strong cellular immunity in vivo, and the resulting antigen-specific T lymphocytes home mainly to dLNs rather than spleens of the responder animals.

### Broadly cross-binding property of serological IgG induced by MAP-based DNA vaccine candidates encoding heterodimeric RBDs

The heterodimeric RBD approach was designed to broaden the spectrum of immune protection against antigen-matched and antigen-mismatched SARS-CoV-2 VOCs. To evaluate the cross-reactivity of MAP^Adv^-DNA-generated antibodies, antisera from mice immunized with MAP-1002, MAP-1003 or MAP-131 were analysed in ELISAs against a panel of 6 recombinant RBDs including RBD^WT^, RBD^Beta^, RBD^Delta^, RBD^BA1^, RBD^BA5^ and RBD^SARS^. Antisera from pWT-immunized mice were included as control. The endpoint dilution RBD-binding titers of serological IgG from the 4 immunization groups are compared in Table 1. Titration curves of the corresponding serum samples are presented in **supplemental Fig. S2.** Here RBD^WT^ and RBD^Delta^ can be regarded as “antigen-mismatched” viral antigens, as they are not encoded by any of the 3 MAP-based DNA vaccine candidates. Interestingly, both these two recombinant RBDs were strongly bound by serum IgG from the MAP-1002, MAP-1003 and MAP-131 groups (average endpoint dilution titers 486,000–1,458,000). Since late 2021, Omicron subvariants BA.4 and BA.5 have been circulating globally and gradually substituted their predecessors BA.1 and BA.2. Mutations in the BA.4 and BA.5 S proteins led to resistance for humoral immune responses induced by early SARS-CoV-2 infection and vaccination [5–11]. Interestingly, both recombinant RBD^BA1^ and RBD^BA5^ were strongly bound by serological IgG of the MAP-DNA-vaccinated mice (average endpoint dilution titers 378,000–486,000). Meanwhile, genetic distance between the immunizing DNA and coating RBD antigens did impact the cross-binding ELISA results. For example, RBD^Beta^-binding titers of the MAP-1003 and MAP-131 (both RBD^Beta^-encoding) antisera were over 3 times that of the MAP-1002 (non-RBD^Beta^-encoding) antisera. RBD^SARS^-binding titer of the MAP-1003 (non-RBD^SARS^-encoding) antisera was less than 1% of the MAP-1002 and MAP-131 (both RBD^SARS^-encoding) antisera. It is also of importance to note that pWT-generated antisera did not bind RBD^BA1^, RBD^BA5^, or RBD^SARS^, and their binding titers to RBD^WT^ and RBD^Delta^ were some 2–10 folds lower compared to that of the MAP-DNA groups. These data support the idea that the RBD chimera approach can improve immunogenicity and help to expand the cross-reaction spectra of COVID-19 vaccines.

**Table 1.** Comparison of RBD cross-binding titers of serum IgG in BALB/c mice following immunization with MAP-based DNA vaccines or pWT/IM + EP^a^.

Coating Ags^b^	MAP-1002^c^	MAP-1003^c^	MAP-131^c^	pWT/IM + EP^c^
RBD^WT^	100	160.30 ± 13.09	344.60 ± 29.53	40.97 ± 6.15
RBD^Beta^	100	313.27 ± 9.69	329.02 ± 19.31	10.53 ± 0.71
RBD^Delta^	100	73.89 ± 4.13	88.97 ± 5.40	<10
RBD^BA1^	100	255.45 ± 25.33	74.38 ± 9.12	<5
RBD^BA4/5^	100	128.67 ± 5.32	174.75 ± 21.96	<5
RBD^SARS^	100	<5	76.98 ± 4.82	<1

^a^ Serum samples from BALB/c mice, collected 14 days after boost immunization with MAP-DNA or pWT/IM + EP, were individually titrated against recombinant RBDs in ELISAs. The resulting titration curves, shown in supplemental Fig.S2, were used to compare RBD-binding IgG titers between the groups with readings of the MAP-1002 group normalized as 100. Data represent mean ± SD of individual serum IgG titers of each group relative to the mean MAP-1002 readings.

^b^ ELISA plates were pre-coated with 2 μg/ml recombinant RBD^WT^, RBD^Delta^, RBD^BA1^, RBD^BA4/5^, or RBD^SARS^.

^c^ Groups of BALB/c mice (*n* = 5) were vaccinated twice with MAP-1002, MAP-1003, MAP-131, or 20 μg pWT/IM + EP on days 0 and 14.

### Cross-neutralization Abs induced by MAP-based DNA vaccines

**Figure 6.**
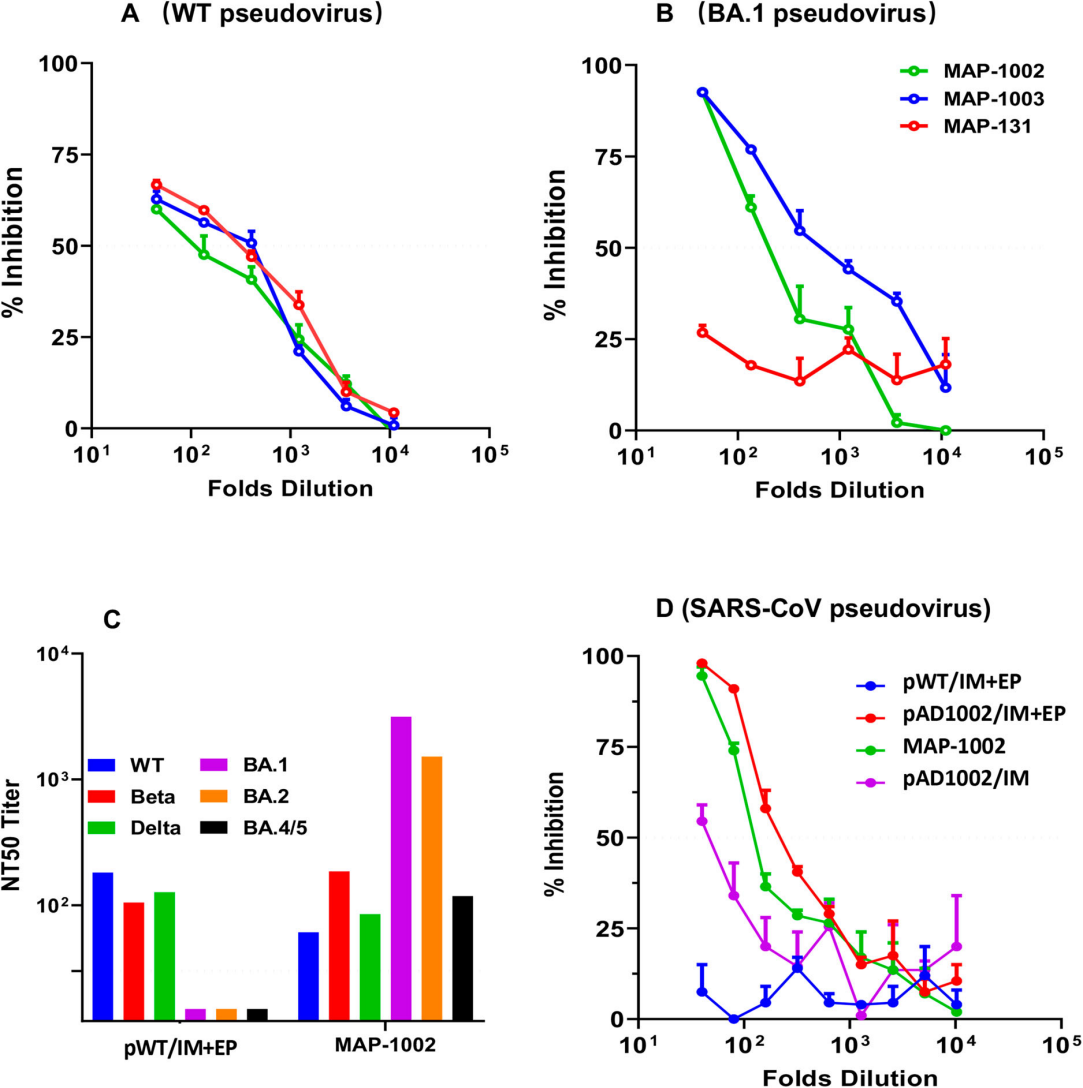
Broad spectrum neutralization of mouse antibodies induced by MAP- DNA vaccination. (**A, B**) BALB/c mouse serum samples collected 14 days post boost immunization with MAP-1002, MAP-1003 or MAP-131 were tested for ability to block mimic infection of hACE2-expressing HEK293T cells by pseudoviruses displaying the S protein of WT SARS-CoV-2 or Omicron BA.1 subvariant. The results are expressed as percent inhibition of infection. (**C**) Antisera from BALB/c mice immunized with either MAP-1002 or pWT/IM + EP were tested for neutralization of pseudoviruses representing WT SARS-CoV-2, Beta, Delta, Omicron BA.1, BA.2 or BA.4/5. The results are expressed as NT50 titers. (**D**) Antisera from BALB/c mice immunized with MAP-1002, pAD1002/IM, pAD1002/IM + EP or pWT/IM + EP were compared for ability to block mimic infection of hACE2-expressing HEK293T cells by SARS-CoV-1 pseudovirus. The results are expressed as percent inhibition of infection. Data are means ± SEM.

Generation of NAbs is known to be crucial for protecting people from virus infection. NAb levels are highly predictive of immune protection from symptomatic SARS-CoV-2 infection in humans [14,15]. It was therefore of importance to ascertain if the high titer RBD-binding Abs induced by MAP-DNA vaccination in model animals possess virus neutralization capability. Firstly, we employed pseudoviruses displaying recombinant S protein of WT SARS-CoV-2, or Omicron subvariant BA.1, to mimic infection of hACE2-expressing HEK293T cells. For WT SARS-CoV-2 pseudovirus blocking, antisera from mice immunized with either MAP-1003, or MAP-1002, or MAP-131 were similarly effective (Figure 6(A)). For Omicron BA.1 pseudovirus blocking, however, MAP-131 antisera were significantly poorer compared to MAP-1003 and MAP-1002 antisera (Figure 6(B)), which is underlined by the fact that construct pADV131 does not encode Omicron RBD and that RBD^BA1^-binding titer of the MAP-131 antisera was lower than that of the MAP-1002 and MAP-1003 antisera (Table 1).

To gain further insight into the neutralization spectra of DNA vaccine-generated NAbs, we next compared MAP-1002 and pWT/IM + EP antisera for ability to block mimic infection of hACE2-transgenic HEK293T cells by a set of pseudoviruses displaying the S protein of WT SARS-CoV-2 or variant Beta, Delta, or Omicron subvariant BA.1, BA.2 or BA.4/5. As shown in Figure 6(C), MAP-1002 antisera neutralized all 6 pseudoviruses, albeit BA.4/5 neutralization titer was relatively lower than that against BA.1 and BA.2. By contrast, pWT/IM + EP antisera neutralized WT, Beta and Delta, but not any of the 3 Omicron pseudoviruses. Additionally, mouse antisera induced by MAP-1002 or pAD1002/IM + EP, but not pWT/IM + EP, were able to neutralize SARS-CoV-1 pseudovirus (Figure 6(D)). Mouse antisera induced by IM pAD1002 vaccination without EP assistance showed only marginal blocking effect in parallel experiments. These results provide proof of concept evidence that MAP^Adv^-based DNA vaccines encoding RBD^SARS^-containing chimera may have the potential to provide broad-spectrum protection against SARS-CoV-2 VOCs and other heterologous Sarbecoviruses.

### Protection efficacy of MAP-1002 in a hACE2-transgenic mouse model

**Figure 7.**
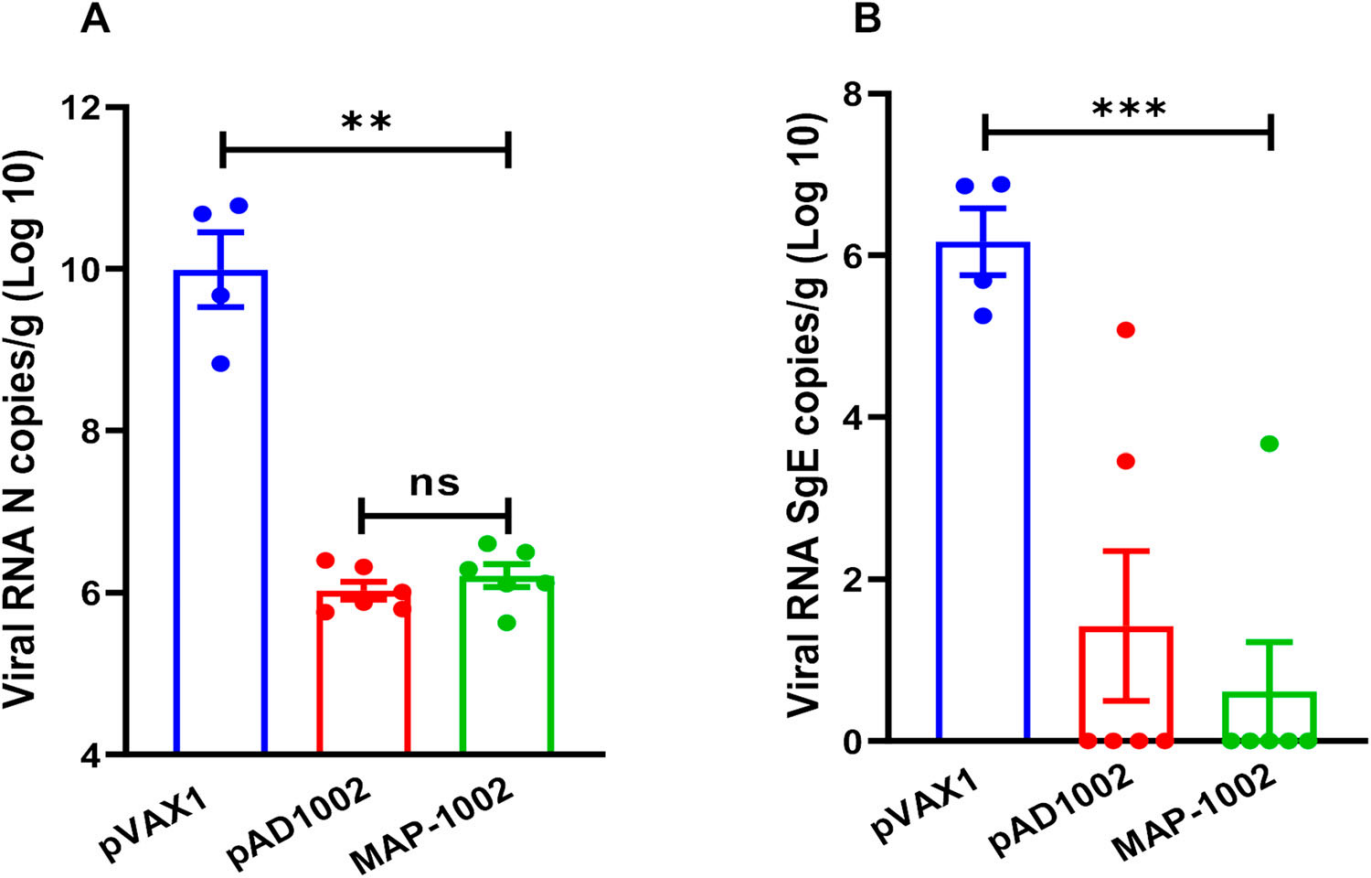
Protection efficacy of MAP-1002. Groups of hACE2-transgenic mice (*n* = 6) were immunized with 2 doses (14 days apart) of MAP-1002, or pAD1002/IM + EP, or pVAX1/IM + EP as sham control. Twenty-one days after boost, the mice were intranasally challenged with 5 × 10^3^ TCID50 of SARS-CoV-2 Omicron BA.1 variant. At 4 dpi, all mice were euthanized and necropsied, and lung samples were collected for virus titration using qPCR for detection of N (**A**) and sgE (**B**) gene sequences of the SARS-CoV-2 BA.1 virus. The results are expressed as viral copy numbers per gram tissue. Data are means ± SEM.

To assess the protective efficacy of MAP-1002, groups of human ACE2-transgenic mice were immunized with 2 doses of MAP-1002, or pAD1002/IM + EP, or pVAX1 as sham control, and then i.n. challenged with viable SARS-CoV-2 Omicron BA.1 variant. No significant change in body weight was observed in any of the groups post-infection (data not shown). At 4 dpi, all mice were euthanized and necropsied, and lung samples were collected for virus titration. As shown in Figure 7(A), the averages of pulmonary viral gRNA were 9.8 × 10^9^ copies/g in the sham group, reduced to 0.9 × 10^6^ copies/g in the pAD1002/IM + EP group, and 1.06 × 10^6^ copies/g in the MAP-1002 group. In line with this, the pulmonary viral sub-genomic RNA (sgRNA) was detected in the sham group with the highest level (average: 1.15 × 10^6^ copies/g), but only detectable in 1 mouse receiving MAP-1002 and 2 mice receiving pAD1002/IM + EP vaccination with average titers of 0.55 × 10^1^ copies/g, and 1.31 × 10^1^ copies/g, respectively, suggesting near complete control of Omicron BA.1 viral replication in pAD1002- and MAP-1002-vaccinated animals (Figure 7(B)).

## Discussion

Effective vaccines that can provide broad coverage against existing and newly emerging SARS-CoV-2 variants are urgently needed. Previous studies showed that tandem RBD^WT^ homodimer or heterodimeric RBD chimera of SARS-CoV-2 VOCs (RBD^WT/Beta^ and RBD^Delta/BA1^) were able to generate cross-protective immunity in mouse and rhesus monkey models [16,17]. We have shown in the present study that pAD1002 (encoding RBD^SARS/BA1^) and pADV131 (encoding RBD^SARS/Beta^) were by far more immunogenic than pAD1003 (encoding RBD^Beta/BA1^) in terms of ability to induce specific IgG when IM administered without EP assistance (Figure 1(G,H); Figure 4(A–C)). The comparatively more potent immunogenicity of pAD1002 and pAD131 could be attributed to the RBD^SARS^ sequence. Molecular structure AI modelling on pAD1002-encoded polypeptide, presented in **supplemental Fig. S3**, suggests that the RBD^SARS^ and RBD^BA1^ domains are kept apart from each other in solution. This is considerably different from the so-called “bilateral lung-like” structure of RBD^WT/Beta^ previously described by Xu et al. [17]. Presumably, the free-moving flexible structure of RBD^SARS/BA1^ and RBD^SARS/Beta^ allows easier interaction with B cell receptors, thereby enabling more efficient B cell activation and NAb production [36,37].

Recent surge of infections by Omicron BF.7, BQ.1 and XBB.1 subvariants in Western countries caused further concern on COVID-19 pandemic control [38]. We found that antisera from the pAD1002-immunized animals were able to block Omicron BA.1, BA.2 and BA.4/5, but not BF.7 and BQ.1, pseudovirus infections (**supplemental Fig. S4**). Interestingly, when the RBD^BA1^-DNA sequence in pAD1002 was replaced with that encoding RBD^BA5^, the resulting plasmid gained the ability to elicit NAbs against Omicron BF.7 and BQ.1 subvariants [39]. It can be envisaged that further adaptation of the vaccine candidate pAD1002 might be required to cover future emerging SARS-CoV-2 VOCs.

Another conclusion that can be drawn from the present study is that MAP^ADV^ was as effective as, if not better than, IM + EP in enhancing DNA vaccination results in vivo. MAP-1002 significantly outperformed inactivated virus vaccine in eliciting RBD-specific IFN-γ^+^ CD8^+^ CTL cells (Figure 5(A)), and generated T lymphocytes of different homing patterns compared to that induced by electroporated DNA in mice (**supplemental Fig. S1**). In consistence with the high titer pseudovirus neutralization results of MAP-1002 antisera, MAP-1002 effectively protected hACE2-transgenic mice from the challenge of viable Omicron BA.1 variant (Figure 7). These data provide supporting evidence for MAP^ADV^ as a potential choice for DNA vaccine delivery in future translational studies.

Different forms of MAPs have been developed for intradermal delivery of naked DNA plasmids or nanoparticle DNA vaccines against infectious disease or cancer in recent years [29–31]. Despite much progress in this field, however, molecular mechanisms for the effectiveness of MAP-mediated DNA immunization are not yet fully understood. In our hands, Luc gene expression in mice after MAP-Luc administration lasted some 15 days (Figure 3(C)), such a durable presence of intradermally synthesized viral antigens could be a robust cue for intense adaptive immunological responses. The skin layers contain abundant antigen-presenting cells (APCs) such as LCs and dendritic cells (DCs) that play important roles in inducing adaptive immunity [26–28]. Vaccine DNA unloaded from an applied 1 cm^2^ skin patch will directly reach some 100,000 APCs in the dermis layer, which could uptake DNA plasmids and then migrate to draining LNs as matured APCs expressing the vaccine-encoded antigen, thereby triggering strong adaptive immunological responses [40]. It should also be noted that the MAP-delivered Luc gene was expressed in waves. Whether such rhythmical expression of DNA vaccines could augment immunological responses of the hosts remains to be investigated. Another possible, but not necessarily mutually exclusive, explanation for the potent MAP-DNA immunogenicity is that physical stimulation caused by MN penetration activates skin tissue cells to secrete inflammatory cytokines and to endocytose (and subsequently express) the unloaded DNA molecules. Finally, the possibility that excipients used for MAP^ADV^ fabrication played a major role in facilitating the immunogenicity of MAP-delivered genes can be excluded, because ID injection of 20 μg pVAX1-Luc plasmid in 30 μl excipient solution produced weak bioluminescence signals at the injection sites (data not shown).

The successful control of COVID-19 pandemic relies not only on the development of vaccines, but also on the storage, transportation, distribution, and effective administration of vaccines. Currently available inactivated virus, subunit or nucleic acid COVID-19 vaccines must be stored at either 4^0^C or frozen. MAP-based DNA vaccines are comparatively more stable. When MAP-1002 was maintained at 25^0^C for one month, for example, no observable immunogenicity decrease compared to those kept in 4^0^C refrigerator was found in terms of ability to induce specific IgG responses in mice (**supplemental Fig. S5**). Additionally, MAPs offer an easy-to-use, painless and minimally invasive alternative to the traditional vaccination methods by directly deposing vaccines amongst a dense population of key immune cells just below the skin surface. In our animal experiments, 15 min application time was enough for the DNA load in MAP^Adv^ to be delivered to the dermis tissue. Increasing MAP application time from 15 to 30 or 60 min did not enhance IgG titers of the recipient animals (**supplemental Fig. S6**). Ongoing experiments in our laboratory will explore the minimum application time required for MAP-DNA vaccination without compromising the immunization results.

In 2021 WHO listed COVID-19 pandemic, vaccine hesitancy and limited vaccine global accessibility as top challenges to global health. By combining the RBD chimera approach, DNA vaccination and MAP technology, our MAP-based DNA vaccine candidates may address all these challenges. Based upon the set of preliminary data accumulated so far, MAP-1002, and future related adaptation products, could serve as COVID-19 vaccine candidates for further translational studies.

## Supplementary Material

Supplemental Material
