# Supplementary material for: Potent immunogenicity and broad-spectrum protection potential of microneedle array patch-based COVID-19 DNA vaccine candidates encoding dimeric RBD chimera of SARS-CoV and SARS-CoV-2 variants
Source: Emerg Microbes Infect. 2023 May 1;12(1):2202269. doi: 10.1080/22221751.2023.2202269 (PMC10155640; doi:10.1080/22221751.2023.2202269)
Supplement: Supplemental Material [file TEMI_A_2202269_SM6680.docx]

**Supplemental Materials**

**Supplemental Figures S1-S6**

Fig. S1. RBD-specific IFN-γ^+^ cell response induced by electroporated pAD1002.

Fig. S2. Comparison of RBD-binding titers of murine serological IgG.

Fig. S3. Molecular structure AI modeling on pAD1002-encoded polypeptide

Fig. S4. Additional pseudo-virus neutralization assays

Fig. S5. Immunogenicity of MAP-1002 after storage at 4^0^C or 25^0^C.

Fig. S6. IgG responses in BALB/c mice induced by MAP-131 administration.

**Supplemental Files SF-1 & SF-2**

**SF-1**. Complete antigen-encoding DNA sequences of the 3 vaccine constructs

**SF-2**. Complete amino acid sequences of the antigens encoded by the 3 vaccine constructs.

**Supplemental Figures**

**A (dLNs)**

**B (Spleen)**

**Figure S1. RBD-specific IFN-γ^+^ cell response induced by electroporated pAD1002**. Draining LN cells (**A**) and splenocytes (**B**) from BALB/c mice (n=5) that had been twice immunized with MAP-1002 (MAP), or pAD1002/IM+EP, or pAD1002/IM were used in ELISpot analysis of IFN-γ spot-forming cells (SFU) after re-stimulation with pooled 14-mer overlapping RBD^WT^ peptides. LNs and spleens from unimmunized mice were included as negative control (NC). Mitomycin-stimulated splenocytes were included as positive control (PC). The results are shown as IFN-γ SFU per million cells. Data represent mean ± SD (n= 5 biologically independent samples).

**Figure S2. Comparison of RBD-binding titers of murine serological IgG.** Serum samples from BALB/c mice (n=5 per group), collected 14 days after boost immunization with MAP-1002, MAP-1003, MAP-131 or 20 μg pWT/IM+EP, were individually titrated against recombinant RBD of WT, Beta, Delta, Omicron BA.1, BA.4/5 of SARS-CoV-2, or SARS-CoV RBD, in ELISAs using HRP-labeled anti-mouse IgG for detection. The results (mean ± SD) are shown as OD (450-650 nm).


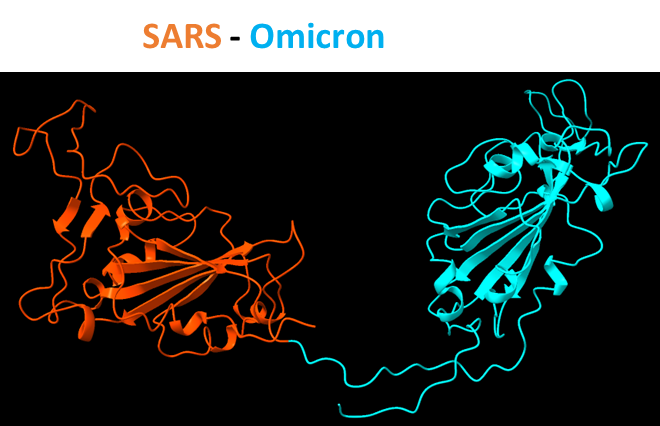


**Figure S3 Molecular structure AI modeling of the pAD1002-encoded polypeptide.** Here the RBD^SARS^ and RBD^BA.1^ domains are kept apart from each other in solution, unlikely to form “bilateral-lung”-like structures of the WT and WT-Beta chimeric SARS-CoV-2 RBD-dimers reported by Xu et al. (17).

**Figure S4. Additional pseudo-virus neutralization assays**

BALB/c mouse (**A**) and rabbit (**B**) serum samples, collected 21 days post boost immunization with MAP-1002, were tested for ability to block mimic infection of ACE2-expressing HEK293T cells by pseudo-viruses displaying S protein of SARS-CoV-2 prototype (WT), Beta, Delta or Omicron BA.1, BA.2, BA.4/5, or BF.7. (**C**) Antisera from BALB/c mice twice immunized with inactivated virus vaccine (Inact), MAP-1002, pVAX-1, or pAD1016 were tested for neutralization of pseudo-typed virus of SARS-CoV-2 variant BQ.1. A serum sample from unimmunized mice was included as negative control (NMS). The results are expressed as NT50 (means ± SEM), and horizontal dashed lines indicate the limit of detection.

**A**

**B**

**Figure S5. Immunogenicity of MAP-1002 after storage at 4^0^C or 25^0^C.** Sample of MAP-1002 were stored at 4^0^C or 25^0^C for 30 days and then used to immunize BALB/c mice (two doses with fortnight interval). Serum samples from the vaccinated animals 14 days after prime and boost immunizations were titrated against recombinant RBD^WT^ in ELISA using HRP-labeled anti-mouse IgG for detection (n=5 biologically independent samples). Sera from mice vaccinated with 20 μg pAD1002/IM+EP (two doses with fortnight interval) were included as controls. Data represent mean ± SD of (**A**) OD_450-650nm_ and (**B**) calculated endpoint dilution titers of IgG.

**Figure S6. IgG responses in BALB/c mice induced by MAP-131 administration.** MAP-131 patches were applied to the shaved skin surface of BALB/c mice with thumb pressure and allowed to stay for 15, 30 or 60 min before removal. Serum samples from the vaccinated animals, collected on days 35, 63, 77 and 90 after primary immunization, were titrated against recombinant RBD^WT^ in ELISA. The results are expressed as endpoint dilution titers of RBD^WT^-specific serological IgG. Data represent mean ± SD (n= 5 biologically independent samples).

**Supplemental Files**

**Supplemental File SF-1. Complete antigen-encoding DNA sequences in the 3 plasmid constructs**

1. **pAD1002**

GACTCTTCGCGATGTACGGGCCAGATATACGCGTTGACATTGATTATTGACTAGTTATTAATAGTAATCAATTACGGGGTCATTAGTTCATAGCCCATATATGGAGTTCCGCGTTACATAACTTACGGTAAATGGCCCGCCTGGCTGACCGCCCAACGACCCCCGCCCATTGACGTCAATAATGACGTATGTTCCCATAGTAACGCCAATAGGGACTTTCCATTGACGTCAATGGGTGGACTATTTACGGTAAACTGCCCACTTGGCAGTACATCAAGTGTATCATATGCCAAGTACGCCCCCTATTGACGTCAATGACGGTAAATGGCCCGCCTGGCATTATGCCCAGTACATGACCTTATGGGACTTTCCTACTTGGCAGTACATCTACGTATTAGTCATCGCTATTACCATGGTGATGCGGTTTTGGCAGTACATCAATGGGCGTGGATAGCGGTTTGACTCACGGGGATTTCCAAGTCTCCACCCCATTGACGTCAATGGGAGTTTGTTTTGGCACCAAAATCAACGGGACTTTCCAAAATGTCGTAACAACTCCGCCCCATTGACGCAAATGGGCGGTAGGCGTGTACGGTGGGAGGTCTATATAAGCAGAGCTCTCTGGCTAACTAGAGAACCCACTGCTTACTGGCTTATCGAAATTAATACGACTCACTATAGGGAGACCCAAGCTGGCTAGCGTTTAAACTTAAGCTTGGTACCGAGCTCggatccGCCACCATGTGGTGGCGGCTGTGGTGGCTGCTGCTGCTGCTGCTGCTGCTGTGGCCCATGGTGTGGGCCAACATCACCAACCTGTGCCCCTTCGGCGAGGTGTTCAACGCCACCAAGTTCCCCAGCGTGTACGCCTGGGAGCGGAAGAAGATCAGCAACTGCGTGGCCGACTACAGCGTGCTGTACAACAGCACCTTCTTCAGCACCTTCAAGTGCTACGGCGTGAGCGCCACCAAGCTGAACGACCTGTGCTTCAGCAACGTGTACGCCGACAGCTTCGTGGTGAAGGGCGACGACGTGCGGCAGATCGCCCCCGGCCAGACCGGCGTGATCGCCGACTACAACTACAAGCTGCCCGACGACTTCATGGGCTGCGTGCTGGCCTGGAACACCCGGAACATCGACGCCACCAGCACCGGCAACTACAACTACAAGTACCGGTACCTGCGGCACGGCAAGCTGCGGCCCTTCGAGCGGGACATCAGCAACGTGCCCTTCAGCCCCGACGGCAAGCCCTGCACCCCCCCCGCCCTGAACTGCTACTGGCCCCTGAACGACTACGGCTTCTACACCACCACCGGCATCGGCTACCAGCCCTACCGGGTGGTGGTGCTGAGCTTCGAGCTGCTGAACGCCCCCGCCACCGTGTGCGGCCCCAGAGTACAGCCTACAGAATCTATCGTTCGGTTTCCAAACATTACCAACCTGTGTCCTTTCGACGAGGTGTTTAACGCCACACGGTTCGCCAGCGTGTATGCCTGGAATAGAAAGCGGATCAGCAACTGTGTGGCCGACTACTCCGTGCTGTACAATCTGGCCCCCTTCTTCACATTTAAGTGCTACGGCGTGTCCCCTACAAAGCTGAACGACCTGTGCTTCACAAACGTGTATGCCGATAGCTTCGTGATCCGGGGCGATGAGGTCCGGCAGATCGCTCCTGGCCAGACAGGCAACATTGCCGACTACAACTACAAGCTGCCCGATGACTTCACCGGATGTGTGATAGCCTGGAACAGCAACAAGCTGGATAGCAAGGTGAGCGGCAACTACAACTACCTGTACCGACTGTTTAGAAAGAGCAACCTGAAACCTTTTGAGCGGGACATCAGCACAGAGATCTACCAAGCCGGCAACAAGCCTTGTAACGGCGTGGCCGGCTTCAACTGTTACTTCCCTCTGCGGTCTTACAGCTTCCGGCCTACATACGGCGTGGGACACCAGCCCTATAGAGTGGTGGTGCTGTCATTCGAGCTGCTACATGCCCCTGCCACCGTGTGCGGCCCTAAGAAGTCTACCAACCTCGTGAAGAACAAGTGATAActcgagTCTAGAGGGCCCGTTTAAACCCGCTGATCAGCCTCGACTGTGCCTTCTAGTTGCCAGCCATCTGTTGTTTGCCCCTCCCCCGTGCCTTCCTTGACCCTGGAAGGTGCCACTCCCACTGTCCTTTCCTAATAAAATGAGGAAATTGCATCGCATTGTCTGAGTAGGTGTCATTCTATTCTGGGGGGTGGGGTGGGGCAGGACAGCAAGGGGGAGGATTGGGAAGACAATAGCAGGCATGCTGGGGATGCGGTGGGCTCTATGGCTTCTACTGGGCGGTTTTATGGACAGCAAGCGAACCGGAATTGCCAGCTGGGGCGCCCTCTGGTAAGGTTGGGAAGCCCTGCAAAGTAAACTGGATGGCTTTCTCGCCGCCAAGGATCTGATGGCGCAGGGGATCAAGCTCTGATCAAGAGACAGGATGAGGATCGTTTCGCATGATTGAACAAGATGGATTGCACGCAGGTTCTCCGGCCGCTTGGGTGGAGAGGCTATTCGGCTATGACTGGGCACAACAGACAATCGGCTGCTCTGATGCCGCCGTGTTCCGGCTGTCAGCGCAGGGGCGCCCGGTTCTTTTTGTCAAGACCGACCTGTCCGGTGCCCTGAATGAACTGCAAGACGAGGCAGCGCGGCTATCGTGGCTGGCCACGACGGGCGTTCCTTGCGCAGCTGTGCTCGACGTTGTCACTGAAGCGGGAAGGGACTGGCTGCTATTGGGCGAAGTGCCGGGGCAGGATCTCCTGTCATCTCACCTTGCTCCTGCCGAGAAAGTATCCATCATGGCTGATGCAATGCGGCGGCTGCATACGCTTGATCCGGCTACCTGCCCATTCGACCACCAAGCGAAACATCGCATCGAGCGAGCACGTACTCGGATGGAAGCCGGTCTTGTCGATCAGGATGATCTGGACGAAGAGCATCAGGGGCTCGCGCCAGCCGAACTGTTCGCCAGGCTCAAGGCGAGCATGCCCGACGGCGAGGATCTCGTCGTGACCCATGGCGATGCCTGCTTGCCGAATATCATGGTGGAAAATGGCCGCTTTTCTGGATTCATCGACTGTGGCCGGCTGGGTGTGGCGGACCGCTATCAGGACATAGCGTTGGCTACCCGTGATATTGCTGAAGAGCTTGGCGGCGAATGGGCTGACCGCTTCCTCGTGCTTTACGGTATCGCCGCTCCCGATTCGCAGCGCATCGCCTTCTATCGCCTTCTTGACGAGTTCTTCTGAATTATTAACGCTTACAATTTCCTGATGCGGTATTTTCTCCTTACGCATCTGTGCGGTATTTCACACCGCATACAGGTGGCACTTTTCGGGGAAATGTGCGCGGAACCCCTATTTGTTTATTTTTCTAAATACATTCAAATATGTATCCGCTCATGAGACAATAACCCTGATAAATGCTTCAATAATAGCACGTGCTAAAACTTCATTTTTAATTTAAAAGGATCTAGGTGAAGATCCTTTTTGATAATCTCATGACCAAAATCCCTTAACGTGAGTTTTCGTTCCACTGAGCGTCAGACCCCGTAGAAAAGATCAAAGGATCTTCTTGAGATCCTTTTTTTCTGCGCGTAATCTGCTGCTTGCAAACAAAAAAACCACCGCTACCAGCGGTGGTTTGTTTGCCGGATCAAGAGCTACCAACTCTTTTTCCGAAGGTAACTGGCTTCAGCAGAGCGCAGATACCAAATACTGTCCTTCTAGTGTAGCCGTAGTTAGGCCACCACTTCAAGAACTCTGTAGCACCGCCTACATACCTCGCTCTGCTAATCCTGTTACCAGTGGCTGCTGCCAGTGGCGATAAGTCGTGTCTTACCGGGTTGGACTCAAGACGATAGTTACCGGATAAGGCGCAGCGGTCGGGCTGAACGGGGGGTTCGTGCACACAGCCCAGCTTGGAGCGAACGACCTACACCGAACTGAGATACCTACAGCGTGAGCTATGAGAAAGCGCCACGCTTCCCGAAGGGAGAAAGGCGGACAGGTATCCGGTAAGCGGCAGGGTCGGAACAGGAGAGCGCACGAGGGAGCTTCCAGGGGGAAACGCCTGGTATCTTTATAGTCCTGTCGGGTTTCGCCACCTCTGACTTGAGCGTCGATTTTTGTGATGCTCGTCAGGGGGGCGGAGCCTATGGAAAAACGCCAGCAACGCGGCCTTTTTACGGTTCCTGGGCTTTTGCTGGCCTTTTGCTCACATGTTCTT

1. **pAD1003**

GACTCTTCGCGATGTACGGGCCAGATATACGCGTTGACATTGATTATTGACTAGTTATTAATAGTAATCAATTACGGGGTCATTAGTTCATAGCCCATATATGGAGTTCCGCGTTACATAACTTACGGTAAATGGCCCGCCTGGCTGACCGCCCAACGACCCCCGCCCATTGACGTCAATAATGACGTATGTTCCCATAGTAACGCCAATAGGGACTTTCCATTGACGTCAATGGGTGGACTATTTACGGTAAACTGCCCACTTGGCAGTACATCAAGTGTATCATATGCCAAGTACGCCCCCTATTGACGTCAATGACGGTAAATGGCCCGCCTGGCATTATGCCCAGTACATGACCTTATGGGACTTTCCTACTTGGCAGTACATCTACGTATTAGTCATCGCTATTACCATGGTGATGCGGTTTTGGCAGTACATCAATGGGCGTGGATAGCGGTTTGACTCACGGGGATTTCCAAGTCTCCACCCCATTGACGTCAATGGGAGTTTGTTTTGGCACCAAAATCAACGGGACTTTCCAAAATGTCGTAACAACTCCGCCCCATTGACGCAAATGGGCGGTAGGCGTGTACGGTGGGAGGTCTATATAAGCAGAGCTCTCTGGCTAACTAGAGAACCCACTGCTTACTGGCTTATCGAAATTAATACGACTCACTATAGGGAGACCCAAGCTGGCTAGCGTTTAAACTTAAGCTTGGTACCGAGCTCggatccGCCACCATGTGGTGGCGGCTGTGGTGGCTGCTGCTGCTGCTGCTGCTGCTGTGGCCCATGGTGTGGGCCAGAGTACAGCCTACAGAATCTATCGTTCGGTTTCCAAACATTACCAACCTGTGTCCTTTCGGCGAGGTGTTTAACGCCACACGGTTCGCCAGCGTGTATGCCTGGAATAGAAAGCGGATCAGCAACTGTGTGGCCGACTACTCCGTGCTGTACAATAGCGCCAGCTTCTCTACATTTAAGTGCTACGGCGTGTCCCCTACAAAGCTGAACGACCTGTGCTTCACAAACGTGTATGCCGATAGCTTCGTGATCCGGGGCGATGAGGTCCGGCAGATCGCTCCTGGCCAGACAGGCAACATTGCCGACTACAACTACAAGCTGCCCGATGACTTCACCGGATGTGTGATAGCCTGGAACAGCAACAACCTGGATAGCAAGGTGGGCGGCAACTACAACTACCTGTACCGACTGTTTAGAAAGAGCAACCTGAAACCTTTTGAGCGGGACATCAGCACAGAGATCTACCAAGCCGGCTCTACCCCTTGTAACGGCGTGAAGGGCTTCAACTGTTACTTCCCTCTGCAGTCTTACGGATTCCAGCCTACATACGGCGTGGGATACCAGCCCTATAGAGTGGTGGTGCTGTCATTCGAGCTGCTACATGCCCCTGCCACCGTGTGCGGCCCTAAGAAGTCTACCAACCTCGTGAAGAACAAGAGAGTACAGCCTACAGAATCTATCGTTCGGTTTCCAAACATTACCAACCTGTGTCCTTTCGACGAGGTGTTTAACGCCACACGGTTCGCCAGCGTGTATGCCTGGAATAGAAAGCGGATCAGCAACTGTGTGGCCGACTACTCCGTGCTGTACAATCTGGCCCCCTTCTTCACATTTAAGTGCTACGGCGTGTCCCCTACAAAGCTGAACGACCTGTGCTTCACAAACGTGTATGCCGATAGCTTCGTGATCCGGGGCGATGAGGTCCGGCAGATCGCTCCTGGCCAGACAGGCAACATTGCCGACTACAACTACAAGCTGCCCGATGACTTCACCGGATGTGTGATAGCCTGGAACAGCAACAAGCTGGATAGCAAGGTGAGCGGCAACTACAACTACCTGTACCGACTGTTTAGAAAGAGCAACCTGAAACCTTTTGAGCGGGACATCAGCACAGAGATCTACCAAGCCGGCAACAAGCCTTGTAACGGCGTGGCCGGCTTCAACTGTTACTTCCCTCTGCGGTCTTACAGCTTCCGGCCTACATACGGCGTGGGACACCAGCCCTATAGAGTGGTGGTGCTGTCATTCGAGCTGCTACATGCCCCTGCCACCGTGTGCGGCCCTAAGAAGTCTACCAACCTCGTGAAGAACAAGTGATAActcgagTCTAGAGGGCCCGTTTAAACCCGCTGATCAGCCTCGACTGTGCCTTCTAGTTGCCAGCCATCTGTTGTTTGCCCCTCCCCCGTGCCTTCCTTGACCCTGGAAGGTGCCACTCCCACTGTCCTTTCCTAATAAAATGAGGAAATTGCATCGCATTGTCTGAGTAGGTGTCATTCTATTCTGGGGGGTGGGGTGGGGCAGGACAGCAAGGGGGAGGATTGGGAAGACAATAGCAGGCATGCTGGGGATGCGGTGGGCTCTATGGCTTCTACTGGGCGGTTTTATGGACAGCAAGCGAACCGGAATTGCCAGCTGGGGCGCCCTCTGGTAAGGTTGGGAAGCCCTGCAAAGTAAACTGGATGGCTTTCTCGCCGCCAAGGATCTGATGGCGCAGGGGATCAAGCTCTGATCAAGAGACAGGATGAGGATCGTTTCGCATGATTGAACAAGATGGATTGCACGCAGGTTCTCCGGCCGCTTGGGTGGAGAGGCTATTCGGCTATGACTGGGCACAACAGACAATCGGCTGCTCTGATGCCGCCGTGTTCCGGCTGTCAGCGCAGGGGCGCCCGGTTCTTTTTGTCAAGACCGACCTGTCCGGTGCCCTGAATGAACTGCAAGACGAGGCAGCGCGGCTATCGTGGCTGGCCACGACGGGCGTTCCTTGCGCAGCTGTGCTCGACGTTGTCACTGAAGCGGGAAGGGACTGGCTGCTATTGGGCGAAGTGCCGGGGCAGGATCTCCTGTCATCTCACCTTGCTCCTGCCGAGAAAGTATCCATCATGGCTGATGCAATGCGGCGGCTGCATACGCTTGATCCGGCTACCTGCCCATTCGACCACCAAGCGAAACATCGCATCGAGCGAGCACGTACTCGGATGGAAGCCGGTCTTGTCGATCAGGATGATCTGGACGAAGAGCATCAGGGGCTCGCGCCAGCCGAACTGTTCGCCAGGCTCAAGGCGAGCATGCCCGACGGCGAGGATCTCGTCGTGACCCATGGCGATGCCTGCTTGCCGAATATCATGGTGGAAAATGGCCGCTTTTCTGGATTCATCGACTGTGGCCGGCTGGGTGTGGCGGACCGCTATCAGGACATAGCGTTGGCTACCCGTGATATTGCTGAAGAGCTTGGCGGCGAATGGGCTGACCGCTTCCTCGTGCTTTACGGTATCGCCGCTCCCGATTCGCAGCGCATCGCCTTCTATCGCCTTCTTGACGAGTTCTTCTGAATTATTAACGCTTACAATTTCCTGATGCGGTATTTTCTCCTTACGCATCTGTGCGGTATTTCACACCGCATACAGGTGGCACTTTTCGGGGAAATGTGCGCGGAACCCCTATTTGTTTATTTTTCTAAATACATTCAAATATGTATCCGCTCATGAGACAATAACCCTGATAAATGCTTCAATAATAGCACGTGCTAAAACTTCATTTTTAATTTAAAAGGATCTAGGTGAAGATCCTTTTTGATAATCTCATGACCAAAATCCCTTAACGTGAGTTTTCGTTCCACTGAGCGTCAGACCCCGTAGAAAAGATCAAAGGATCTTCTTGAGATCCTTTTTTTCTGCGCGTAATCTGCTGCTTGCAAACAAAAAAACCACCGCTACCAGCGGTGGTTTGTTTGCCGGATCAAGAGCTACCAACTCTTTTTCCGAAGGTAACTGGCTTCAGCAGAGCGCAGATACCAAATACTGTCCTTCTAGTGTAGCCGTAGTTAGGCCACCACTTCAAGAACTCTGTAGCACCGCCTACATACCTCGCTCTGCTAATCCTGTTACCAGTGGCTGCTGCCAGTGGCGATAAGTCGTGTCTTACCGGGTTGGACTCAAGACGATAGTTACCGGATAAGGCGCAGCGGTCGGGCTGAACGGGGGGTTCGTGCACACAGCCCAGCTTGGAGCGAACGACCTACACCGAACTGAGATACCTACAGCGTGAGCTATGAGAAAGCGCCACGCTTCCCGAAGGGAGAAAGGCGGACAGGTATCCGGTAAGCGGCAGGGTCGGAACAGGAGAGCGCACGAGGGAGCTTCCAGGGGGAAACGCCTGGTATCTTTATAGTCCTGTCGGGTTTCGCCACCTCTGACTTGAGCGTCGATTTTTGTGATGCTCGTCAGGGGGGCGGAGCCTATGGAAAAACGCCAGCAACGCGGCCTTTTTACGGTTCCTGGGCTTTTGCTGGCCTTTTGCTCACATGTTCTT

1. **pADV131**

GACTCTTCGCGATGTACGGGCCAGATATACGCGTTGACATTGATTATTGACTAGTTATTAATAGTAATCAATTACGGGGTCATTAGTTCATAGCCCATATATGGAGTTCCGCGTTACATAACTTACGGTAAATGGCCCGCCTGGCTGACCGCCCAACGACCCCCGCCCATTGACGTCAATAATGACGTATGTTCCCATAGTAACGCCAATAGGGACTTTCCATTGACGTCAATGGGTGGACTATTTACGGTAAACTGCCCACTTGGCAGTACATCAAGTGTATCATATGCCAAGTACGCCCCCTATTGACGTCAATGACGGTAAATGGCCCGCCTGGCATTATGCCCAGTACATGACCTTATGGGACTTTCCTACTTGGCAGTACATCTACGTATTAGTCATCGCTATTACCATGGTGATGCGGTTTTGGCAGTACATCAATGGGCGTGGATAGCGGTTTGACTCACGGGGATTTCCAAGTCTCCACCCCATTGACGTCAATGGGAGTTTGTTTTGGCACCAAAATCAACGGGACTTTCCAAAATGTCGTAACAACTCCGCCCCATTGACGCAAATGGGCGGTAGGCGTGTACGGTGGGAGGTCTATATAAGCAGAGCTCTCTGGCTAACTAGAGAACCCACTGCTTACTGGCTTATCGAAATTAATACGACTCACTATAGGGAGACCCAAGCTGGCTAGCGTTTAAACTTAAGCTTGGTACCGAGCTCggatccGCCACCATGTGGTGGCGGCTGTGGTGGCTGCTGCTGCTGCTGCTGCTGCTGTGGCCCATGGTGTGGGCCAACATCACCAACCTGTGCCCCTTCGGCGAGGTGTTCAACGCCACCAAGTTCCCCAGCGTGTACGCCTGGGAGCGGAAGAAGATCAGCAACTGCGTGGCCGACTACAGCGTGCTGTACAACAGCACCTTCTTCAGCACCTTCAAGTGCTACGGCGTGAGCGCCACCAAGCTGAACGACCTGTGCTTCAGCAACGTGTACGCCGACAGCTTCGTGGTGAAGGGCGACGACGTGCGGCAGATCGCCCCCGGCCAGACCGGCGTGATCGCCGACTACAACTACAAGCTGCCCGACGACTTCATGGGCTGCGTGCTGGCCTGGAACACCCGGAACATCGACGCCACCAGCACCGGCAACTACAACTACAAGTACCGGTACCTGCGGCACGGCAAGCTGCGGCCCTTCGAGCGGGACATCAGCAACGTGCCCTTCAGCCCCGACGGCAAGCCCTGCACCCCCCCCGCCCTGAACTGCTACTGGCCCCTGAACGACTACGGCTTCTACACCACCACCGGCATCGGCTACCAGCCCTACCGGGTGGTGGTGCTGAGCTTCGAGCTGCTGAACGCCCCCGCCACCGTGTGCGGCCCCAGAGTACAGCCTACAGAATCTATCGTTCGGTTTCCAAACATTACCAACCTGTGTCCTTTCGGCGAGGTGTTTAACGCCACACGGTTCGCCAGCGTGTATGCCTGGAATAGAAAGCGGATCAGCAACTGTGTGGCCGACTACTCCGTGCTGTACAATAGCGCCAGCTTCTCTACATTTAAGTGCTACGGCGTGTCCCCTACAAAGCTGAACGACCTGTGCTTCACAAACGTGTATGCCGATAGCTTCGTGATCCGGGGCGATGAGGTCCGGCAGATCGCTCCTGGCCAGACAGGCAACATTGCCGACTACAACTACAAGCTGCCCGATGACTTCACCGGATGTGTGATAGCCTGGAACAGCAACAACCTGGATAGCAAGGTGGGCGGCAACTACAACTACCTGTACCGACTGTTTAGAAAGAGCAACCTGAAACCTTTTGAGCGGGACATCAGCACAGAGATCTACCAAGCCGGCTCTACCCCTTGTAACGGCGTGAAGGGCTTCAACTGTTACTTCCCTCTGCAGTCTTACGGATTCCAGCCTACATACGGCGTGGGATACCAGCCCTATAGAGTGGTGGTGCTGTCATTCGAGCTGCTACATGCCCCTGCCACCGTGTGCGGCCCTAAGAAGTCTACCAACCTCGTGAAGAACAAGTGATAActcgagTCTAGAGGGCCCGTTTAAACCCGCTGATCAGCCTCGACTGTGCCTTCTAGTTGCCAGCCATCTGTTGTTTGCCCCTCCCCCGTGCCTTCCTTGACCCTGGAAGGTGCCACTCCCACTGTCCTTTCCTAATAAAATGAGGAAATTGCATCGCATTGTCTGAGTAGGTGTCATTCTATTCTGGGGGGTGGGGTGGGGCAGGACAGCAAGGGGGAGGATTGGGAAGACAATAGCAGGCATGCTGGGGATGCGGTGGGCTCTATGGCTTCTACTGGGCGGTTTTATGGACAGCAAGCGAACCGGAATTGCCAGCTGGGGCGCCCTCTGGTAAGGTTGGGAAGCCCTGCAAAGTAAACTGGATGGCTTTCTCGCCGCCAAGGATCTGATGGCGCAGGGGATCAAGCTCTGATCAAGAGACAGGATGAGGATCGTTTCGCATGATTGAACAAGATGGATTGCACGCAGGTTCTCCGGCCGCTTGGGTGGAGAGGCTATTCGGCTATGACTGGGCACAACAGACAATCGGCTGCTCTGATGCCGCCGTGTTCCGGCTGTCAGCGCAGGGGCGCCCGGTTCTTTTTGTCAAGACCGACCTGTCCGGTGCCCTGAATGAACTGCAAGACGAGGCAGCGCGGCTATCGTGGCTGGCCACGACGGGCGTTCCTTGCGCAGCTGTGCTCGACGTTGTCACTGAAGCGGGAAGGGACTGGCTGCTATTGGGCGAAGTGCCGGGGCAGGATCTCCTGTCATCTCACCTTGCTCCTGCCGAGAAAGTATCCATCATGGCTGATGCAATGCGGCGGCTGCATACGCTTGATCCGGCTACCTGCCCATTCGACCACCAAGCGAAACATCGCATCGAGCGAGCACGTACTCGGATGGAAGCCGGTCTTGTCGATCAGGATGATCTGGACGAAGAGCATCAGGGGCTCGCGCCAGCCGAACTGTTCGCCAGGCTCAAGGCGAGCATGCCCGACGGCGAGGATCTCGTCGTGACCCATGGCGATGCCTGCTTGCCGAATATCATGGTGGAAAATGGCCGCTTTTCTGGATTCATCGACTGTGGCCGGCTGGGTGTGGCGGACCGCTATCAGGACATAGCGTTGGCTACCCGTGATATTGCTGAAGAGCTTGGCGGCGAATGGGCTGACCGCTTCCTCGTGCTTTACGGTATCGCCGCTCCCGATTCGCAGCGCATCGCCTTCTATCGCCTTCTTGACGAGTTCTTCTGAATTATTAACGCTTACAATTTCCTGATGCGGTATTTTCTCCTTACGCATCTGTGCGGTATTTCACACCGCATACAGGTGGCACTTTTCGGGGAAATGTGCGCGGAACCCCTATTTGTTTATTTTTCTAAATACATTCAAATATGTATCCGCTCATGAGACAATAACCCTGATAAATGCTTCAATAATAGCACGTGCTAAAACTTCATTTTTAATTTAAAAGGATCTAGGTGAAGATCCTTTTTGATAATCTCATGACCAAAATCCCTTAACGTGAGTTTTCGTTCCACTGAGCGTCAGACCCCGTAGAAAAGATCAAAGGATCTTCTTGAGATCCTTTTTTTCTGCGCGTAATCTGCTGCTTGCAAACAAAAAAACCACCGCTACCAGCGGTGGTTTGTTTGCCGGATCAAGAGCTACCAACTCTTTTTCCGAAGGTAACTGGCTTCAGCAGAGCGCAGATACCAAATACTGTCCTTCTAGTGTAGCCGTAGTTAGGCCACCACTTCAAGAACTCTGTAGCACCGCCTACATACCTCGCTCTGCTAATCCTGTTACCAGTGGCTGCTGCCAGTGGCGATAAGTCGTGTCTTACCGGGTTGGACTCAAGACGATAGTTACCGGATAAGGCGCAGCGGTCGGGCTGAACGGGGGGTTCGTGCACACAGCCCAGCTTGGAGCGAACGACCTACACCGAACTGAGATACCTACAGCGTGAGCTATGAGAAAGCGCCACGCTTCCCGAAGGGAGAAAGGCGGACAGGTATCCGGTAAGCGGCAGGGTCGGAACAGGAGAGCGCACGAGGGAGCTTCCAGGGGGAAACGCCTGGTATCTTTATAGTCCTGTCGGGTTTCGCCACCTCTGACTTGAGCGTCGATTTTTGTGATGCTCGTCAGGGGGGCGGAGCCTATGGAAAAACGCCAGCAACGCGGCCTTTTTACGGTTCCTGGGCTTTTGCTGGCCTTTTGCTCACATGTTCTT

**Supplemental File SF-2. Amino acid sequences of the antigens encoded by the 3 vaccine constructs**

1. **pAD1002-encoded amino acid sequence**

Leader sequence—SARS-CoV-1 RBD-SARS-CoV-2 Omicron BA.1 RBD

MWWRLWWLLLLLLLLWPMVWANITNLCPFGEVFNATKFPSVYAWERKKISNCVADYSVLYNSTFFSTFKCYGVSATKLNDLCFSNVYADSFVVKGDDVRQIAPGQTGVIADYNYKLPDDFMGCVLAWNTRNIDATSTGNYNYKYRYLRHGKLRPFERDISNVPFSPDGKPCTPPALNCYWPLNDYGFYTTTGIGYQPYRVVVLSFELLNAPATVCGPRVQPTESIVRFPNITNLCPFDEVFNATRFASVYAWNRKRISNCVADYSVLYNLAPFFTFKCYGVSPTKLNDLCFTNVYADSFVIRGDEVRQIAPGQTGNIADYNYKLPDDFTGCVIAWNSNKLDSKVSGNYNYLYRLFRKSNLKPFERDISTEIYQAGNKPCNGVAGFNCYFPLRSYSFRPTYGVGHQPYRVVVLSFELLHAPATVCGPKKSTNLVKNK*

1. **pAD1003-encoded amino acid sequence**

Leader sequence—SARS-CoV-2 Beta RBD-SARS-CoV-2 Omicron BA.1 RBD

MWWRLWWLLLLLLLLWPMVWARVQPTESIVRFPNITNLCPFGEVFNATRFASVYAWNRKRISNCVADYSVLYNSASFSTFKCYGVSPTKLNDLCFTNVYADSFVIRGDEVRQIAPGQTGNIADYNYKLPDDFTGCVIAWNSNNLDSKVGGNYNYLYRLFRKSNLKPFERDISTEIYQAGSTPCNGVKGFNCYFPLQSYGFQPTYGVGYQPYRVVVLSFELLHAPATVCGPKKSTNLVKNKRVQPTESIVRFPNITNLCPFDEVFNATRFASVYAWNRKRISNCVADYSVLYNLAPFFTFKCYGVSPTKLNDLCFTNVYADSFVIRGDEVRQIAPGQTGNIADYNYKLPDDFTGCVIAWNSNKLDSKVSGNYNYLYRLFRKSNLKPFERDISTEIYQAGNKPCNGVAGFNCYFPLRSYSFRPTYGVGHQPYRVVVLSFELLHAPATVCGPKKSTNLVKNK*

1. **pADV131-encoded amino acid sequence**

Leader sequence—SARS-CoV-1 RBD-SARS-CoV-2 Beta RBD

MWWRLWWLLLLLLLLWPMVWANITNLCPFGEVFNATKFPSVYAWERKKISNCVADYSVLYNSTFFSTFKCYGVSATKLNDLCFSNVYADSFVVKGDDVRQIAPGQTGVIADYNYKLPDDFMGCVLAWNTRNIDATSTGNYNYKYRYLRHGKLRPFERDISNVPFSPDGKPCTPPALNCYWPLNDYGFYTTTGIGYQPYRVVVLSFELLNAPATVCGPRVQPTESIVRFPNITNLCPFGEVFNATRFASVYAWNRKRISNCVADYSVLYNSASFSTFKCYGVSPTKLNDLCFTNVYADSFVIRGDEVRQIAPGQTGNIADYNYKLPDDFTGCVIAWNSNNLDSKVGGNYNYLYRLFRKSNLKPFERDISTEIYQAGSTPCNGVKGFNCYFPLQSYGFQPTYGVGYQPYRVVVLSFELLHAPATVCGPKKSTNLVKNK*
